# Niosomes as Biocompatible Scaffolds for the Multivalent Presentation of Tumor-Associated Antigens (TACAs) to the Immune System

**DOI:** 10.1021/acs.bioconjchem.2c00383

**Published:** 2022-12-15

**Authors:** Silvia Fallarini, Francesco Papi, Federico Licciardi, Francesca Natali, Grazia Lombardi, Francesca Maestrelli, Cristina Nativi

**Affiliations:** † Department of Pharmaceutical Sciences, University of “Piemonte Orientale”, Novara 28100, Italy; ‡ Department of Chemistry, University of Florence, Sesto Fiorentino, Florence 50019, Italy; § CNR-IOM and INSIDE@ILL, c/o OGG, 71 avenue des Martyrs, 38042 Grenoble Cedex 9, France

## Abstract

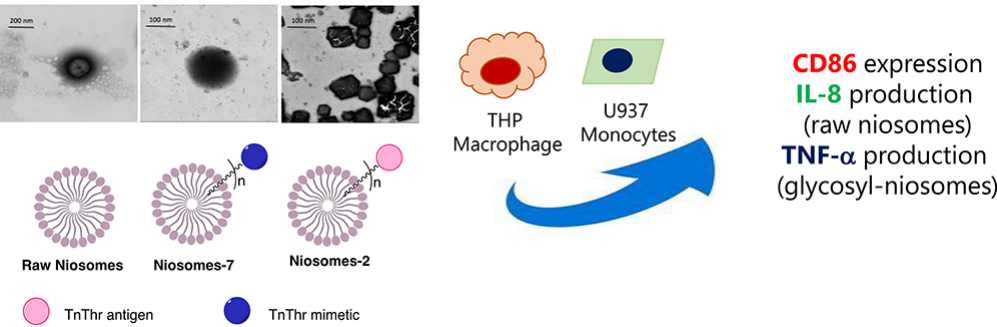

Fully synthetic tumor-associated carbohydrate antigen (TACA)-based vaccines are a promising strategy to treat cancer. To overcome the intrinsic low immunogenicity of TACAs, the choice of the antigens’ analogues and multivalent presentation have been proved to be successful. Here, we present the preparation, characterization, and *in vitro* screening of niosomes displaying multiple copies of the mucin antigen TnThr (niosomes-**7**) or of TnThr mimetic **1** (niosomes-**2**). Unprecedentedly, structural differences, likely related to the carbohydrate portions, were observed for the two colloidal systems. Both niosomal systems are stable, nontoxic and endowed with promising immunogenic properties.

## Introduction

**Figure 1:**
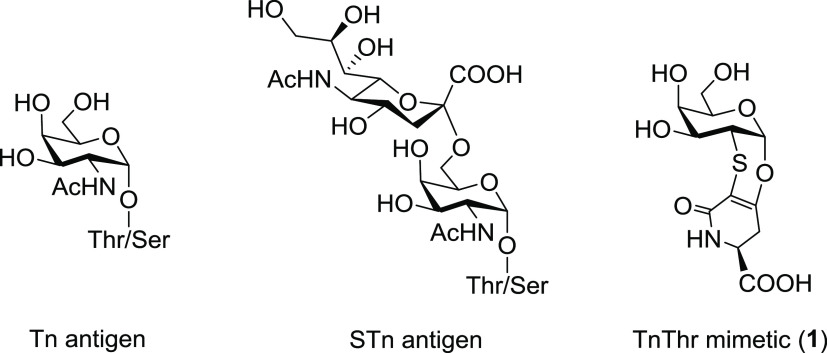
Structure of α-Tn and STn native antigens and of TnThr mimetic **1**.

Glycosylation is one of the most important posttranslational modifications of proteins and it is of pivotal relevance for cell growth, differentiation, and signaling. Thus, it is not surprising that aberrant cell transformations are characterized by abnormal protein glycosylations.^1^ Cancer cells, for example, are marked by significant modifications in terms of carbohydrate expression. These altered saccharides, known as tumor-associated carbohydrate antigens (TACAs), often characteristic of specific cancer cells, can be used to differentiate cancer cells from normal cells and are exploited as therapeutic targets.^2^ This is the case of mucins (MUCs), a glycoprotein family displaying under physiological conditions long, branched *O*-glycosidic chains, but found truncated and oversimplified in tumor cells.^3^ MUC1-related TACAs are widely studied tumoral markers that have been identified in almost all human epithelial adenocarcinomas.^4^ Among them, α-Tn (α-GalNAc-O-Ser/Thr) and sialyl Tn (STn) antigens have been detected in up to 90% of human breast and ovary cancers, thus becoming objects of great interest as therapeutic targets (Figure 1).^4,5^ In particular, they have widely been studied as antigens for the development of promising candidate vaccines against cancer.^6,7^ However, TACAs, including Tn and STn, do not elicit strong humoral responses, being T cell-independent antigens that require a multivalent presentation on immunogenic carrier molecules to elicit T cell activation and long-lasting immune responses.

A common strategy to boost the antigenicity of TACAs consists in linking isolated TACAs to proteins like ovalbumin (OVA), tetanus toxoid (TT), or detoxified diphtheria toxin (CRM_197_) as adjuvant carriers.^8^ Following a different approach, fully synthetic vaccine candidates have been prepared by conjugating Tn and STn antigens to a peptide T cell epitope and a Toll-like receptor (TLR) agonist as the internal adjuvant.^7^ More recently, a fully synthetic vaccine candidate was proposed by formulating multiple copies of an α-GalNAc conjugate into liposomes.^9^ Liposome formulations have been reported to elicit more reproducible glycan immunity with respect to conventional glycoconjugate vaccines, displaying the same saccharide antigen; therefore, antigenic glycolipids assembled into liposomes of different sizes have successfully been used to immunize mice.^10^ However, although a wide variety of immunogenic constructs have proved promising in preclinical animal studies, the few that reached clinical trials were disappointing in terms of disease progression and increasing the survival.^7^

Along with the low intrinsic immunogenicity of TACAs, one major drawback affecting TACA-based cancer vaccines’ efficacy is the sensitivity of the glycosidic linkages to endogenous glycosidases, which reduces their *in vivo* bioavailability.^11−13^ Consequently, TACA analogues or mimetics have been developed to obtain enzymatically more stable structures preserving B-cell immunogenicity.^14−19^

**Scheme 1:**
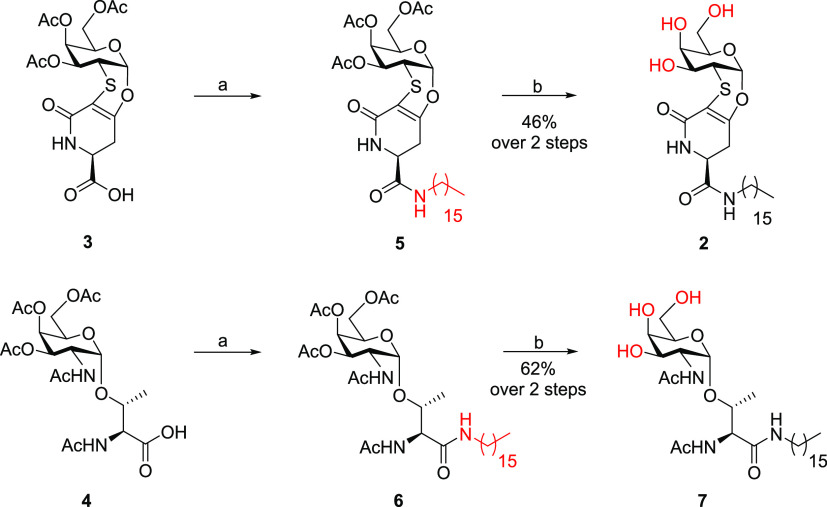
Synthesis of HexadecylAmine Glycolipids **2** and **7**; (a) HAD, HBTU, DIPEA, dry CH_2_Cl_2_, rt, 1 h; (b) NH_3_ (4 M in MeOH), rt, 2 h

In this framework, some years ago we developed the thioether-bridged TnThr mimetic **1** (see Figure 1), which preserves the pharmacophoric conformation of the native antigen with an increased *in vivo* stability.^20^ A remarkable immunomodulatory activity was observed in *in vivo* trials when mimetic **1** was multivalently presented to the immune system.^21,22^ Relying on the structural properties of mimetic **1** and on our previous experience regarding antigenic glycolipids assembled into vesicles,^23^ in this work we functionalized **1** with a lipid chain and the glycolipid **2** so obtained (Scheme 1) was assembled into niosomes. Niosomes are nanovesicles, more stable, safe, and less expensive than liposomes, obtained with synthetic surfactants that can be functionalized with precise ligands and selectively recognized by specific receptors.^24,25^ The aim of this work was the preparation of niosomes decorated with multiple copies of TnThr mimetic **1** and to screen *in vitro* their immunogenic properties with respect to niosomes functionalized with the native TnThr antigen and to monovalent glycolipids **2** and **7** (Scheme 1).

## Results and Discussion

### Synthesis of Glycolipids **2** and **7**

To incorporate TnThr mimetic **1** and the natural TnThr antigen into niosomes, the two α-galactosides were functionalized with a hexadecyl alkyl chain (Scheme 1). The covalent linkage of acetylated TnThr mimetic **3**(26) and of acetylated native TnThr **4**(27) with hexadecylamine (HAD) was run under condensation conditions in dry CH_2_Cl_2_ as solvent, at room temperature, in the presence of HBTU and DIPEA. After deacetylation of crude derivatives **5** and **6** with NH_3_ (4 M in CH_3_OH), deprotected glycolipids **2** and **7** were isolated, respectively.

### Niosome Characterization and Stability Studies

Niosomes were prepared using a thin-layer evaporation paddle, which was a partial modification of a previous method.^24,25^ A detailed description of the niosomes’ preparation is provided in Supporting Information. The particle size, polydispersion index (PDI), and ζ-potential of niosomes, freshly prepared and after reconstitution, are reported in Table S1. After reconstitution, raw niosomes (blank) and niosomes-**2** have a size below 150 nm in accordance with our previous work;^24^ thus, no effect was observed on the size of niosomes loaded with glycolipid **2**. Conversely, larger vesicles were obtained upon loading with glycolipid **7**. The dimensions’ reduction observed for the three batches after reconstitution is probably the consequence of the highest-energy sonication performed after the lyophilization process (Table S1, Supporting Information).

**Figure 2:**
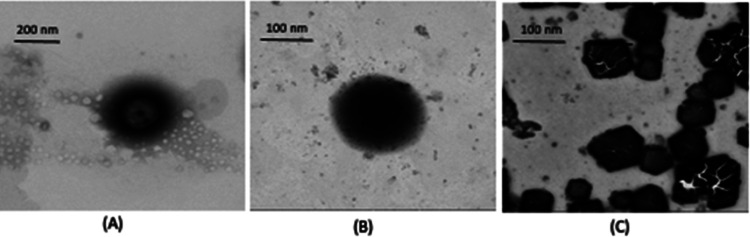
Scanning transmission electron microscopy (STEM) micrographs obtained with FIB-SEM Microscope Gaia 3 of (A) raw niosomes (blank) (mag. 259kx); (B) niosomes-**7** (mag. 182kx); and (C) niosomes-**2** (mag. 236kx).

The morphological examination performed on the three colloidal batches is reported in Figure 2.

As shown in Figure 2, raw niosomes (A) evidenced a spherical shape and a smaller dimension with respect to niosomes charged with glycolipid **7** (B), in accordance with the DLS findings. In addition, a difference between raw and charged niosomes can be observed; as a matter of fact, while raw niosomes (blank) showed a lighter dark core and a darker layer, niosomes charged with the native TnThr-based glycolipid **7** (niosomes-**7**, B) present a homogeneous and dark surface, very likely due to the presence of TnThr residues. Instead, niosomes charged with the TnThr mime-based glycolipid **2** (niosomes-**2**, C) showed a remarkably different morphology. The presence of mimetic **1** residues led to the acquisition of ordered hexagonal structures, probably due to the higher rigidity of this molecule.

**Figure 3:**
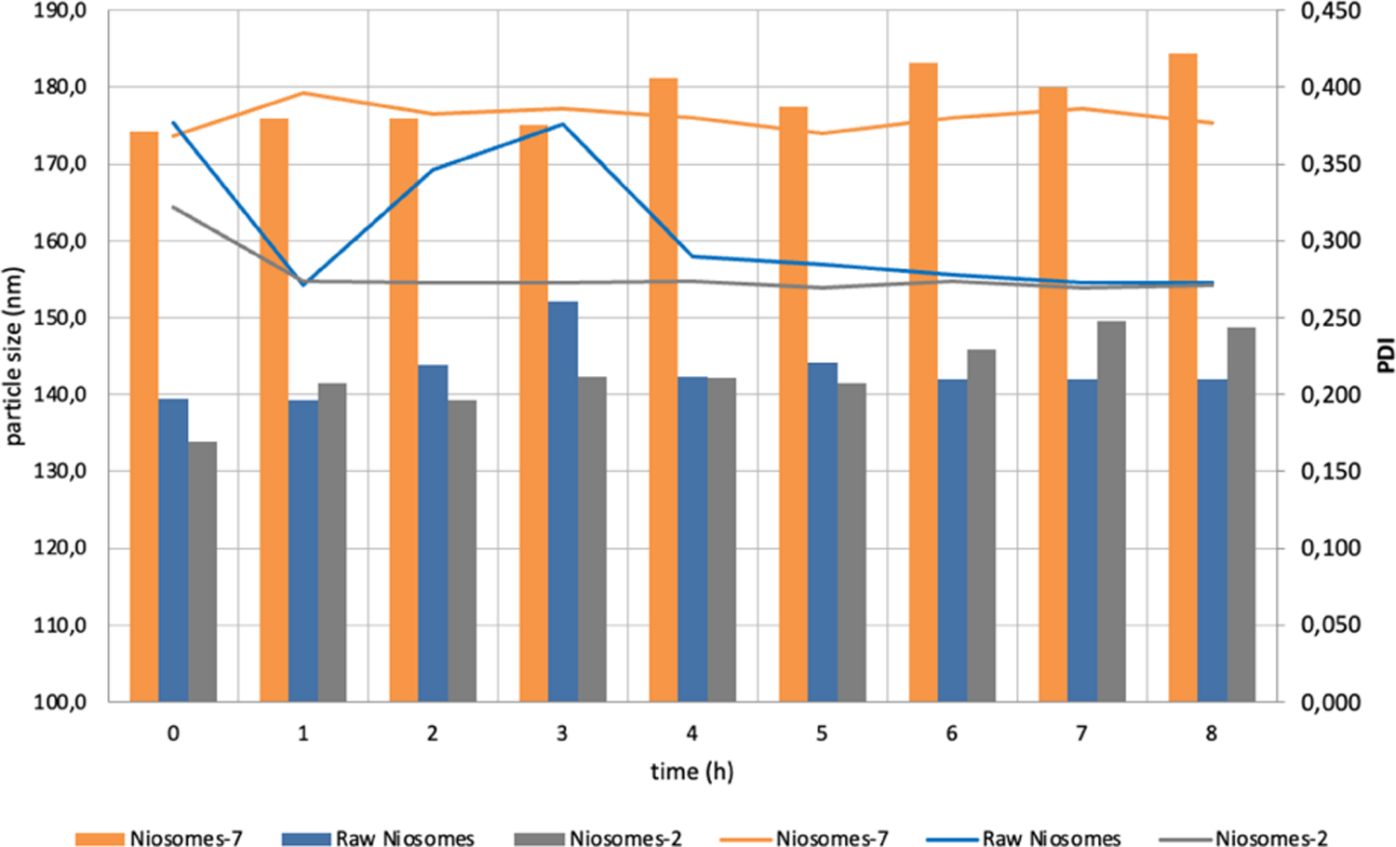
Stability studies performed for 8 h in HBSS (Hanks’ Buffered Saline Solution) at 37 °C, reported in terms of particle size (bars) and PDI (lines).

Niosomes’ stability studies were performed in HBSS at 37 °C to trigger the *in vivo* behavior and the results are summarized in Figure 3.

A good stability of modified niosomes was observed compared to the blank (*p* < 0.05). Both the particle size and PDI of the three batches were maintained during the 8 h of the test, thus confirming the niosomes’ stability in the medium.

### Niosome Internal Dynamics

The internal macromolecular dynamics was investigated using elastic incoherent neutron scattering, providing access to local motions falling within ∼100 ps time scale window (see Supporting Information for details). Clear evidence of the acquired stiffness (higher *k* value) of niosomes-**2** local dynamics can be appreciated (Figure S1). The effect is more pronounced when the niosomes are loaded with glycolipid **7** (niosome-**7**). This is mainly assigned to the very local dynamical processes and in particular to the lipid chain defect motions and the rotational diffusion about the lipid molecular axis, which occur at the 10^–11^ s time scale (thus, within the time scale accessible with the used spectrometer).

### Cell Viability

Human THP-1 monocytic and human U937 pro-monocytic cell lines were differentiated into macrophages M0 by incubation in the presence of phorbol 12-myristate 13-acetate (PMA) (150 and 100 nM, respectively). Macrophage differentiation with PMA resulted in a slight reduction of cell viability that was not further significantly reduced by both interferon (IFN)-γ and lipopolysaccharide (LPS) (Figures S3A,C and S4).

Treatment with the compounds did not reduce the cell viability at any of the concentrations tested when compared with the PMA-treated THP-1 or U937 cells (Figure S3B,D). These results clearly indicate that all compounds were biocompatible and could be used for further studies.

### Monocyte Differentiation into Macrophages

#### Morphological Characterization

As previously observed,^28^ macrophage differentiation with PMA is associated with a reduction in the nucleo/cytoplasmic ratio due to an expansion in cytoplasmic volume (Figure S5, Supporting Information) as well as due to an increase in granularity caused by an increase of some organelles. PMA treatment induces an increase in the forward side scatter/side scatter (FSC/SSC) parameter of both THP-1 and U937 cells, a typical marker associated with macrophage differentiation. To obtain M1 polarization, M0 macrophages (PMA-treated THP-1 or U937 cells) were treated with IFN-γ (20 ng/mL) or LPS (0.5 μg/mL) for 24 h.^29,30^ The increased FSC/SSC parameters were maintained also under these treatments. Moreover, the treatment with the tested compounds preserves the change induced in the FSC/SSC parameters, suggesting their ability to sustain M0 differentiation (Figure S5).

**Figure 4:**
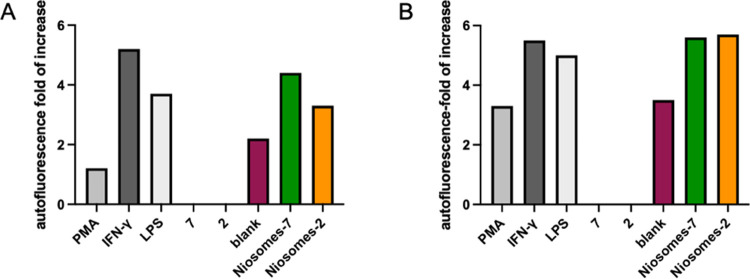
Effects of the test compounds on THP-1 and U937 cell autofluorescence. Autofluorescence fold increase of THP-1 cells (A) and U937 cells (B) treated for 24 h with 150 and 100 nM of PHA, respectively, and of M0 treated (24 h) with 0.5 mg/mL of LPS or 20 ng/mL of IFN-γ or 1 mg/mL of the tested compounds. The results represent the mean ± standard error of the mean (SEM) of at least three independent experiments.

The PMA-induced differentiation of THP-1 and U937 toward M0 macrophages was also confirmed by another distinguishing feature of differentiation: the increase in autofluorescence that is low in monocytes, but amplified in macrophage differentiated cells. As shown in Figures 4 and S5, the autofluorescence slightly increases in PMA-treated cells (+1.2- and +3.3-fold change for THP-1 and U937 cells, respectively), but it reaches the highest value with IFN-γ or LPS treatment (+5.2- and +3.7-fold change, respectively, for THP-1 cells and +5.5- and +5-fold change, respectively, for U937). The treatment with glycolipids **7** or **2** did not increase the autofluorescence when compared with PMA-treated cells, while raw niosomes slightly increased this parameter (+2.2- and +3.5-fold change for THP-1 and U937, respectively). A higher increase was observed when M0 macrophages were treated with the functionalized niosome (+4.4- and +3.3-fold change for niosomes-**7** and niosomes-**2**, respectively); higher levels were also measured in U937 cells (+5.6- and +5.7-fold change for niosomes-**7** and niosomes-**2**, respectively). These results clearly showed that functionalized niosomes induce a differentiation very similar to that induced by IFN-γ or LPS treatment and that the effect is not cell dependent but compound dependent.

#### Phenotypic Characterization

**Figure 5:**
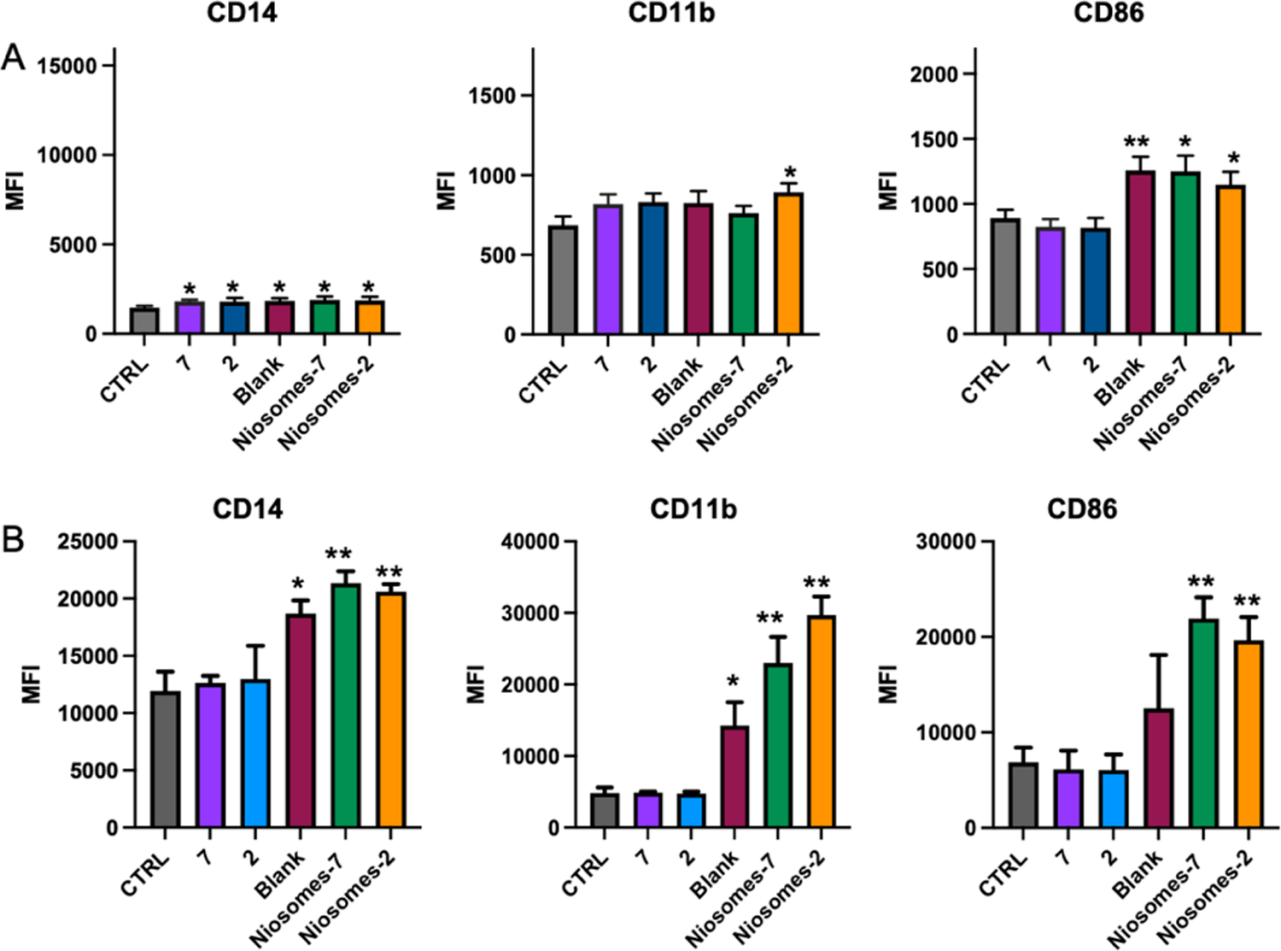
Effects of compounds on marker surface expression on differentiated THP-1 and U937 cells. THP-1 (A) and U937 (B) levels of expression of CD14, CD11b, and CD86 on M0 treated (24 h) with **7**, **2**, blank, niosomes-**7**, and niosomes-**2**. The results represent the mean ± SEM of at least three independent experiments. * ≤0.05 treated *vs* control (CTRL); ** ≤0.01 treated *vs* control (CTRL); *** ≤0.001 treated *vs* control.

To confirm that the changes in FSC/SSC and autofluorescence parameters reflect a differentiation toward macrophages, the analysis of surface markers was performed. As shown in Figure S6 (see Supporting Information), PMA as well as IFN-γ or LPS treatment of THP-1 or U937 induces a change in marker expression. A higher increase in CD14 expression was observed for IFN-γ-treated M0 (+9.2-fold increase for THP-1 cells and +2.1-fold increase for U937 cells) when compared to PMA- or LPS-treated cells (+7.1- and +1.6-fold increase, respectively, in THP-1 cells and + 1.3- and +1.7-fold increase, respectively, for U937 cells). Similarly, an increase was observed for CD11b expression that reached higher dimensions for both IFN-γ- and LPS-treated M0 (+1.3-fold and +1.4-fold change, respectively, for THP-1 cells and + 2.8- and +3.9-fold increase, respectively, for U937 cells). In contrast, the increase in CD86 expression was observed only for IFN-γ- and LPS-treated M0 cells (+2.3-fold and +2-fold change, respectively, for THP-1 cells and +2.7- and +2.3-fold increase, respectively, for U937 cells) (Figure S6). Since the CD86 marker is a M1 marker, these results clearly confirm that both IFN-γ and LPS treatments induce the M1 polarization. As shown in Figure 5, also treatment with the tested compounds induced a change in marker expression. Each compound induced a similar increase in CD14 expression in THP-1 cells (Figure 5A), while only blank and functionalized niosomes were able to induce an upregulation of CD14 in the U937 cell line (Figure 5B). In contrast, a significant increase in CD11b expression was observed only after niosomes**-2** treatment in THP-1 cells (Figure 5A), while both blank and functionalized niosomes increased the CD11b levels in U937 cells (Figure 5B). Of note, treatment of M0 macrophages with raw or loaded niosomes induced an increase in CD86 expression that was not induced by monovalent glycolipids **2** and **7**, suggesting that niosomes are able to induce an M1 polarization; in particular, in U937 cells, only functionalized niosomes induced CD86 upregulation.

#### Cytokine Profile

**Figure 6:**
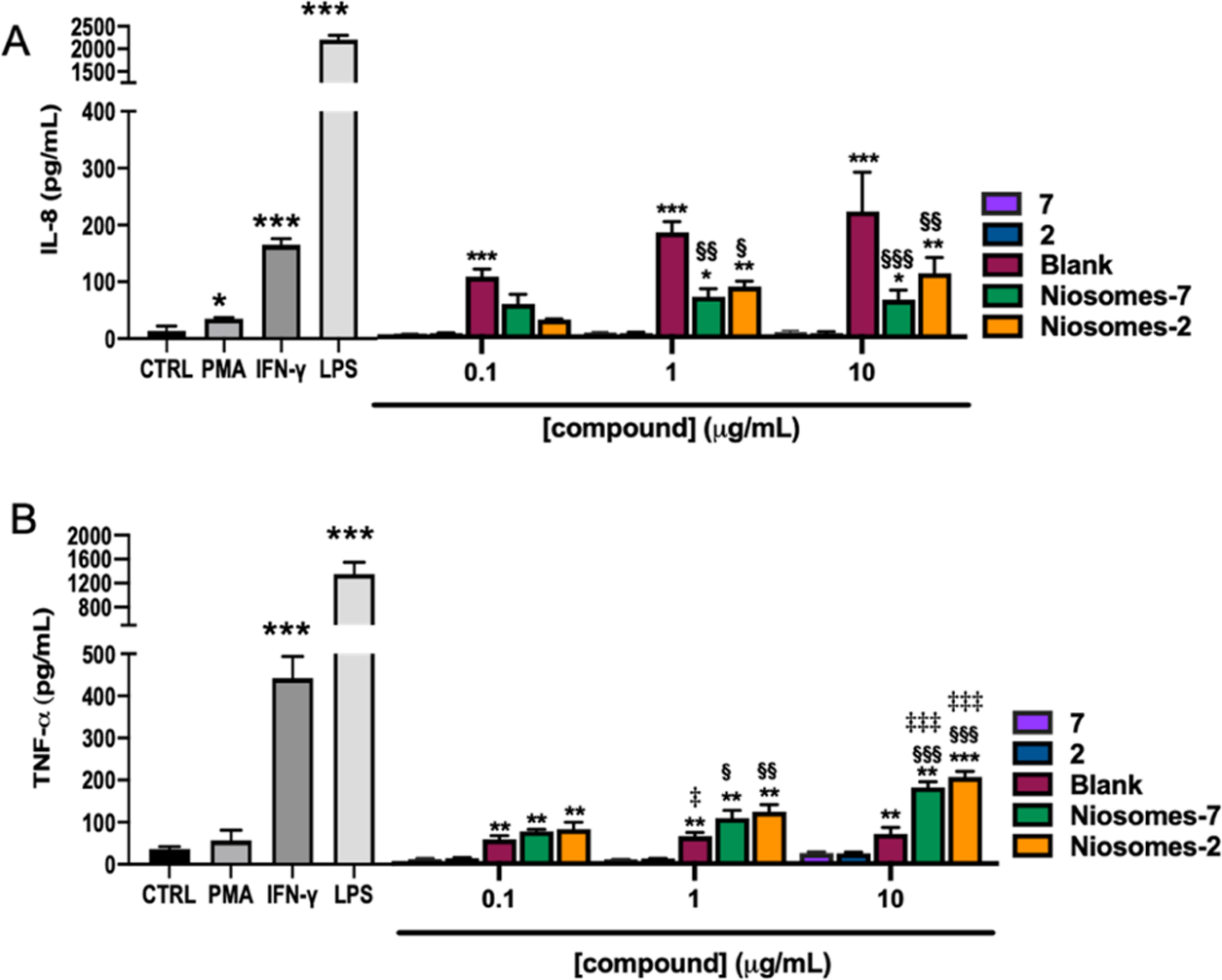
Effects of the tested compounds on cytokine secretion in differentiated THP-1 cells. PMA-differentiated THP-1 cells were treated with 0.5 μg/mL of LPS or 20 ng/mL of IFN-γ or 1 μg/mL of the tested compounds for 48 h; the cell culture medium was harvested, and IL-8 (A) and TNF-α (B) levels were measured by ELISA assay. The results represent the mean ± SEM of at least three independent experiments. * ≤0.05 treated *vs* control (CTRL); ** ≤0.01 treated *vs* control (CTRL); *** ≤0.001 treated *vs* control (CTRL); § ≤0.05 treated *vs* Blank; §§ ≤0.01 treated *vs* Blank; §§§ ≤0.001 treated *vs* Blank; ‡ ≤0.05 compound low concentration vs compound high concentration; ‡‡ ≤0.01 compound low concentration *vs* compound high concentration; ‡‡‡ ≤0.001 compound low concentration *vs* compound high concentration.

Monocytes toward macrophage differentiation correlated with change in cytokine production. In particular, THP-1 and U937 differentiation correlates mainly with an increase in IL-1β, TNF-α, IL-8, and IL-6 gene expression and protein production. In particular, in THP-1 cells, a higher cytokine release was observed for TNF-α and IL-8.^29^ PMA treatment induces a slight increase in cytokine production, but a significant increase was observed after treatment with both IFN-γ and LPS. The higher increase for both IL-8 and TNF-α was obtained by LPS treatment (Figure 6). Glycolipids **2** and **7** did not induce cytokine release at any concentration tested. In THP-1 cells, raw niosomes (blank) induced both IL-8 and TNF-α release in a concentration-dependent manner, which is significant when TNF-α is considered. The release of IL-8 from raw niosome (blank)-treated cells was higher when compared with the glycosyl niosomes at all concentrations tested (Figure 6).

**Figure 7:**
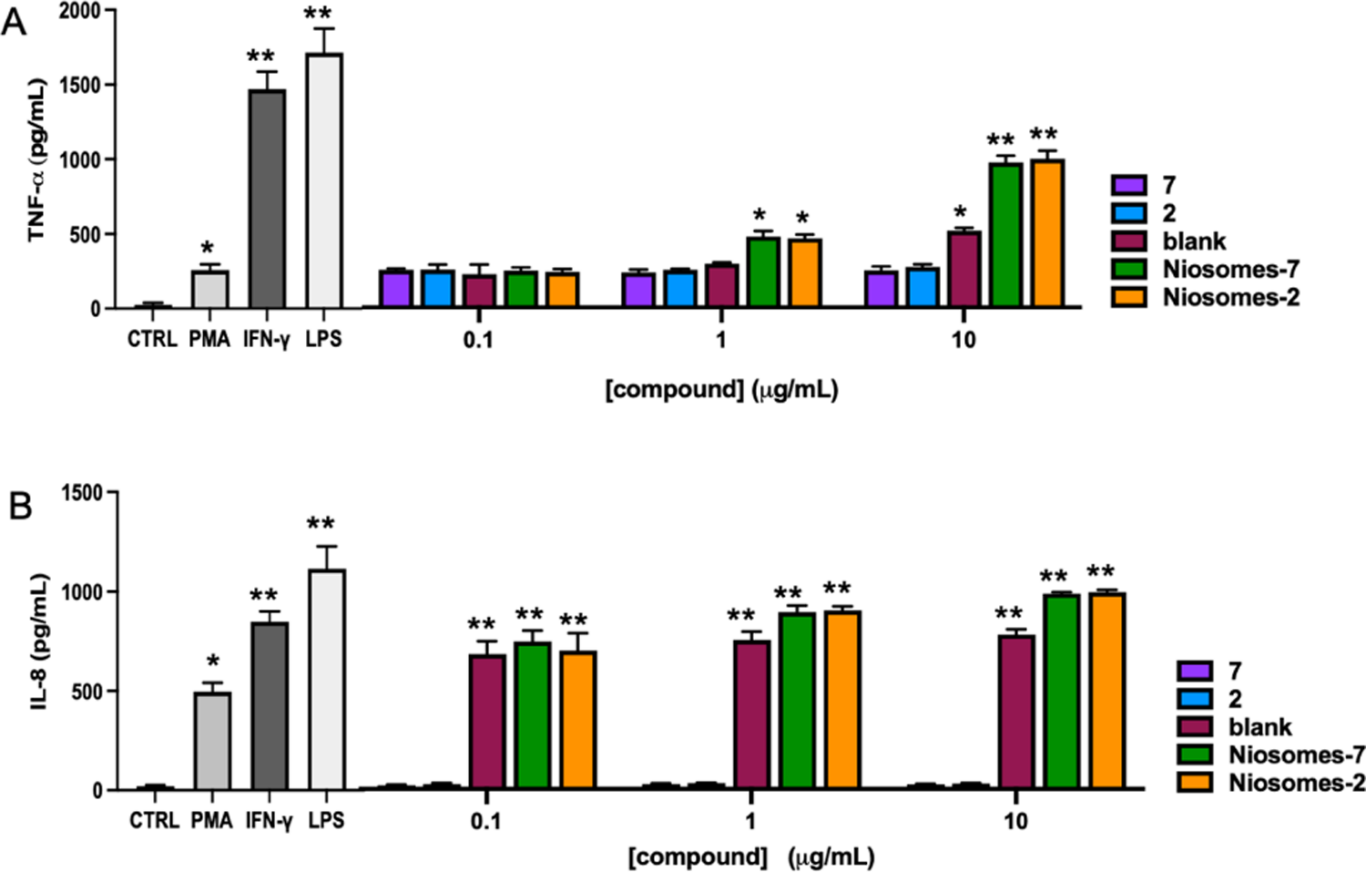
Effects of the tested compounds on cytokine secretion in differentiated U937 cells. PMA-differentiated U937 cells were treated with 0.5 mg/mL of LPS or 20 ng/mL of IFN-g or 1 mg/mL of the tested compounds for 48 h; the cell culture medium was harvested, and IL-8 (A) and TNF-a (B) levels were measured by ELISA assay. The results represent the mean ± SEM of at least three independent experiments. * ≤0.05 treated *vs* control (CTRL); ** ≤0.01 treated *vs* control (CTRL).

However, in U937 cells, blank treatment induced a significant increase of TNF-α release only at 10 μg/mL and induced a not concentration-dependent increase of IL-8 release (Figure 7). Both niosomes-**2** and niosomes-**7** induced a significant concentration-dependent increase of IL-8 and TNF-α release. The TNF-α release by both niosomes-**2** and niosomes-**7** was higher when compared with raw niosomes when tested at 1 and 10 μg/mL. It is worth noting that when THP-1 is considered at 10 μg/mL, niosomes-**2** induced a higher release of both IL-8 (62 *vs* 106 pg/mL for niosomes-**2** and niosomes-**7**, respectively) and TNF-α (170 *vs* 200 pg/mL for niosomes-**2** and niosomes-**7**, respectively) when compared with niosomes-**7**, even though significance was not reached.

#### Hemocompatibility Assays

**Figure 8:**
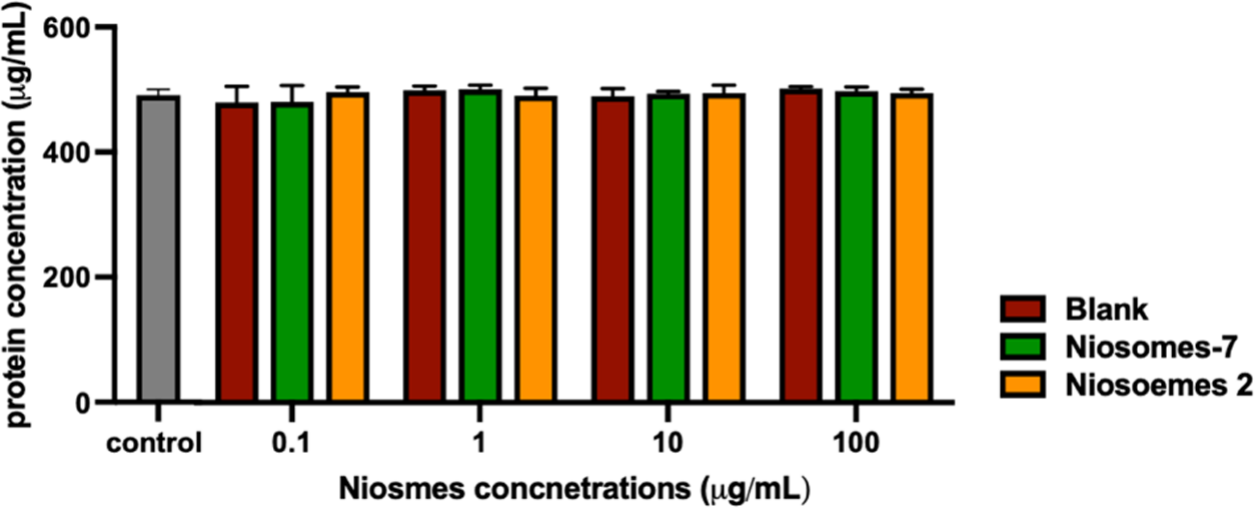
Effects of the tested compounds on the blood protein level. Levels of protein concentrations in the human donor’s peripheral blood incubated with increasing concentrations of niosomes (glycosylated and nonglycosylated). The results represent the mean ± SEM of at least three independent experiments conducted with blood from different donors.

The hemocompatibility tests are usually performed to explore the possible toxic effect or interaction ability of nanomaterials with the hematic components such as plasma protein, leukocytes, lymphocytes, or red blood cells. So, in these assays we have tested the activity of glycosylated niosomes (niosomes-**7** and niosomes-**2**) and the nonglycosylated niosome (blank). The early interaction of niosomes with the blood component was indirectly tested by measuring the protein concentration in the blood. Protein content was evaluated by Bradford assay. After the niosomes’ incubation, the serum protein levels remained similar to that measured in the control for all niosomes and concentrations tested. These results suggest that proteins are not absorbed on the surface of the niosomes (Figure 8).

**Figure 9:**
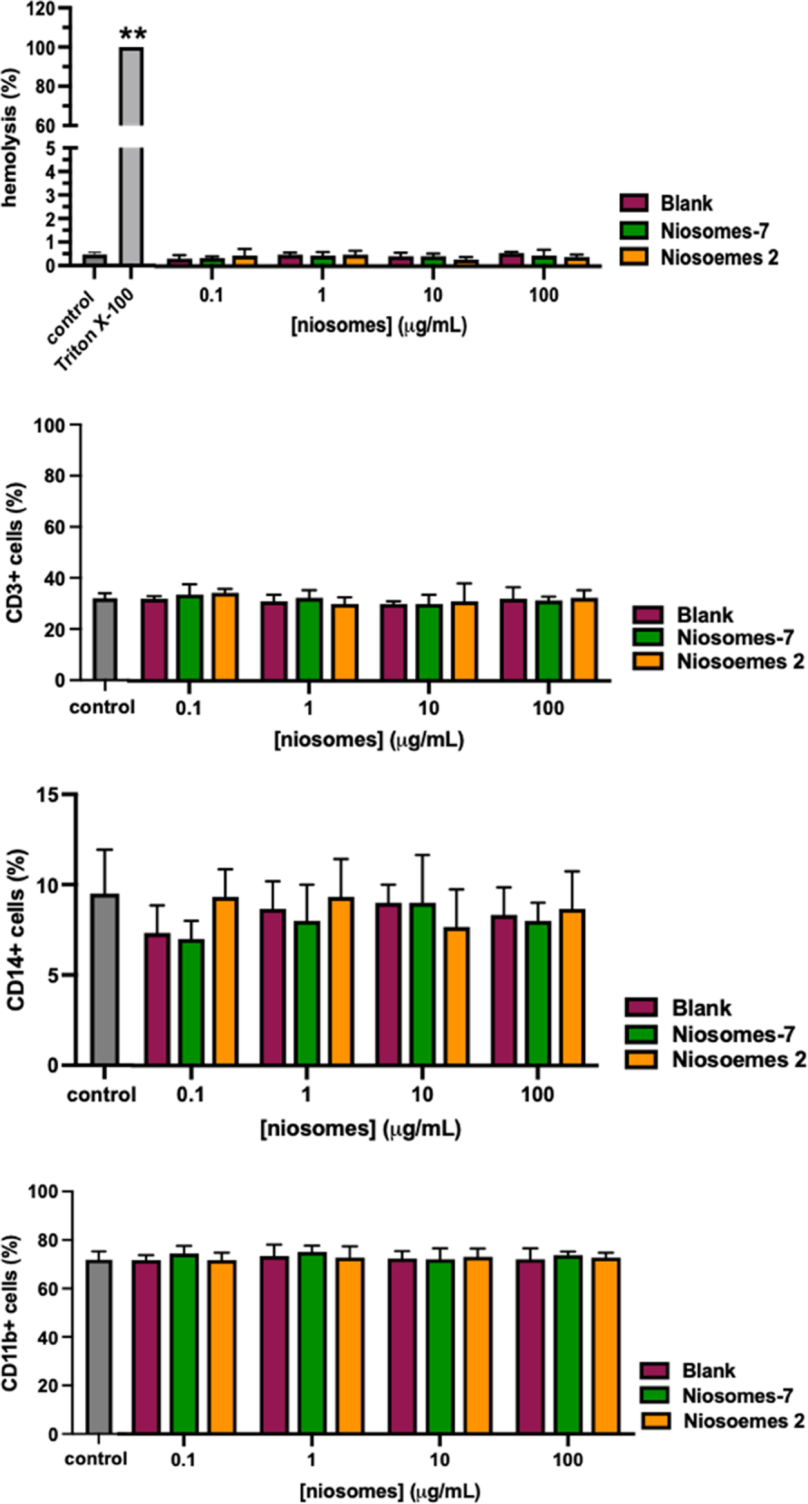
Effects of the tested compounds on blood cell viability. Percentage of red blood cell lysis, of lymphocytes, monocytes, and granulocytes in the human donor’s peripheral blood incubated with increasing concentrations of niosomes (glycosylated or nonglycosylated). The results represent the mean ± SEM of at least three independent experiments conducted with blood from different donors. ** ≤0.01 treated *vs* control.

Subsequently, the cytotoxicity potential of niosomes on the different components of blood was evaluated. All of the niosomes resulted not toxic at all concentrations tested. Indeed, glyco-niosomes and raw niosomes, independently of their conjugation or concentration, induced hemolysis after 4 h of incubation when compared with the control. Moreover, the percentage of viable CD45^+^, CD11b^+^, CD14^+^, and CD3^+^ cells remained unchanged after the treatment with niosomes (Figure 9). These results clearly suggest that all of the synthesized compounds are not toxic.

The multivalent presentation of TACAs to the immune system is a critical issue to break the immune tolerance *vs* carbohydrate antigens. For the multivalent display of the TnThr antigen mimetic **1**, size-defined niosomes were prepared. Mimetic **1** is enzymatically stable and immunogenic and is characterized by a carboxylic hook, which was suitably functionalized for the insertion of a C16 aliphatic chain. The glycolipid **2** obtained was thus used to prepare the corresponding glycosyl niosomes as biocompatible multivalent constructs (niosomes-**2**). Similarly, native Tn antigen was transformed into the glycolipid **7**, which, in turn, was employed to prepare glycosyl niosomes displaying multiple copies of Tn antigen (niosomes-**7**). Niosomes-**2** and niosomes-**7** were fully characterized in terms of size, PDI, zeta potential, and morphology. With respect to raw niosomes, niosomes-**2** and niosomes-**7** are characterized by a smaller size (214.3, 175.8, and 194.4, respectively, see Table S1), a lower PDI (0.412, 0.247, and 0.274), and zeta potential (−51.8, −38.9, and −26.6). After reconstitution, the raw niosomes and niosomes-**2** have, as expected, a size below 150 nm (139.5 and 133.9, respectively, see Table S1), while larger vesicles were obtained in the case of niosomes-**7**. Striking differences were observed for the morphology of niosomes-**2** compared to the other two batches. Although raw niosomes are smaller than niosomes-**7**, both these vesicle systems appear spherical (see Figure 2), in agreement with the DLS finding. Ordered hexagonal structures were instead observed for niosomes-**2**. This phenomenon often occurs for self-assembled porous silica materials and for alamethicin, a rather rigid 20-amino acid peptide with a rod-like structure, when it is inserted in lipidic membranes.^31−33^ The major structural rigidity and the less efficient packing of mimetic **1** residues with respect to native Tn antigen portions might thus account for the glycosyl niosomes’ morphological difference (macroscopic level).

The intriguing morphological data prompted us to investigate glycosyl niosomes’ internal dynamics. Elastic incoherent neutron scattering provided access to microscopic local motions. The elastic intensity data (see Figure S2) clearly showed an acquired stiffness of niosomes-**2** and niosomes-**7** local dynamics compared to raw niosomes (blank). Of note, the effect is more pronounced for niosomes-**7**. Thus, the addition of natural TnThr antigen undoubtedly induced strong changes in the niosome dynamics, reducing the proton local mobility. The different proton local mobilities of the niosomes might be due to the different hydration levels of the sugar residues decorating niosomes-**7** and niosomes-**2**. The more hydrated niosomes-**7** (higher H-bond network) are more rigid at the microscopic level.

The good stability of raw niosomes, niosomes-**2** and niosomes-**7**, along with their lack of toxicity (see Figure S3), clearly proved the biocompatibility of the glycosylated vesicles we propose and the interest in screening their immunomodulating properties *in vitro*. Macrophages represent one of the main players in the immune system. A key feature of these cells is their plasticity and ability to tailor their response depending on microenvironmental signals. Thanks to these skills, macrophages are orchestrating cells, involved in many physiological and pathological processes. Their main role is to sense, through specialized receptors, pathogenic associated molecular pattern (PAMP) and damage-associated molecular pattern (DAMP) signals and initiate the most adequate immune response that involves the release of mediators and the recruitment of other immune cells.^34^ Macrophages are also involved in the generation and orchestration of antitumor immune responses, so we have evaluated *in vitro* the immunomodulatory ability of the new prepared niosomes on macrophages. The models selected are the THP-1 human leukemia monocytic cell line and the U937 human leukemia pro-monocytic cell line, which are widely used models to study the ability of compounds to induce monocytic-derived macrophage differentiation and activation.^35^ As indicated by the morphological characterization,^28^ PMA treatment induced THP-1 and U937 cell differentiation toward M0 unpolarized and inactivated macrophages, and the subsequent treatment of M0 macrophages with LPS and IFN-γ resulted in M1 polarization. Both niosomes-**2** and niosomes-**7** induced both THP-1 and U937 cell morphological changes very close to those observed by treatment with the golden standard IFN-γ or LPS (see Figure 4). No differentiation was recorded upon treatment with monovalent glycolipids **7** or **2**, while an intermedium level of differentiation was reached when M0 macrophages were treated with raw niosomes (blank). The differentiation toward macrophages was confirmed by the analysis of THP-1 and U937 cell surface markers (see Figure 5). In particular, either monovalent glycolipids or niosomes induced a similar increase in CD14 expression on THP-1 (see Figure 5B), a typical macrophage marker^36^ that is present on M0 macrophages, indicating that all compounds are able to sustain M0 differentiation. Of note, treatment of M0 macrophages with raw or loaded niosomes induced an increase in CD86 expression, a typical M1 marker^37^ that was not induced by monovalent glycolipids **2** or **7**. These results suggest that the multivalent presentation obtained by niosomes is able to overcome the less immunogenic potential typical of carbohydrate antigens like Tn antigen and the mimetic **1**.^38^ Interestingly, also the niosomes-related adjuvant properties can be responsible for this result. Indeed, also raw niosomes are able to induce CD86 upregulation at least in the THP-1 cell line. Many researchers exploring the use of niosomes in vaccines have observed that niosomes act as potent adjuvants in both *in vitro* and *in vivo* studies when used with a weakly immunogenic antigen.^39^ In particular, a preferential Th1-mediated immune response is observed, which is fundamental for the therapeutic vaccine activity.^39^ Of note, the adjuvant effect is observed also when the antigen is encapsulated in the niosomes,^35,40^ suggesting that the structure/composition of niosomes is responsible for the adjuvant effect. Finally, the macrophages’ response is the result of the activating signals induced by the species present in the microenvironment. Raw niosomes and glycosylated niosomes possess different surfaces that can interact with different receptors, generating different second signals and responses. This could justify the differences in the markers’ upregulation and cytokine production induced by different compounds. The significant increase in CD11b expression observed only upon treatment with niosomes-**2** in THP-1 cells (see Figure 5A) and the different levels observed in U937 cells treated with raw niosomes or niosomes-**2** and niosomes-**7** (see Figure 5B) further suggest that the surface composition of niosomes could have a predominant effect on the macrophages’ response. Finally, these data confirm that glycosylated niosomes are able to induce M1 polarization, which is the prerequisite to induce a proinflammatory response. Functionally, the M1 macrophages participate in the removal of pathogens during infection and cell debris during tissue damage; the material engulfed is processed and antigen presented in the context of MHC class I molecules to T cells.^34^ The upregulation of CD86 induced by niosomes-**2** and niosomes-**7** clearly suggests their acquisition of ability as antigen-presenting cells, which is particularly important in the case of a tumor therapeutic vaccine.

The other fundamental feature of M1 macrophages is their ability to orchestrate proinflammatory and antitumoral immune responses. To fulfill this task, macrophages produce and release chemokines and cytokines able to tailor an efficient antitumor immune response.^41^ Both raw niosomes and glycosylated niosomes induced an increase in IL-8 production. IL-8 (along with MCP-1, CXCL9, and CXCL10) is a chemokine able to recruit myeloid and lymphoid cells to the inflamed site; the recruitment phase is the first step to mount an efficient immune response since it leads to colocalization of regulator and effector cells.^42,43^ This effect is not observed with glycolipids **2** and **7**, further confirming the adjuvant role of niosomes. The IL-8 release induced by raw niosomes in THP-1 cells is greater than that observed with glycosylated niosomes; even if apparently there is a concentration-dependent cytokine release, the differences are not significant, suggesting that this result can be ascribed to the surface characteristics of niosomes (see Figure 6). The results obtained with raw niosomes in U937 further confirm the surface effect on cell activation and were corroborated by the different levels of IL-8 induced by niosomes-**2** and niosomes-**7** (see Figure 7). Of note, although the release of IL-8 is desirable for early-stage tumors, it is kept low because of its pro-tumorigenic properties.^44^ Therefore, the lower induction of IL-8 shown by niosomes-**2** and niosomes-**7** with respect to LPS is positive. In the same way, raw niosomes and glycosylated niosomes induced an increase in TNF-α production even though at different levels. TNF-α (along with IL-1β) is an important player in tumor diseases mediating relevant processes including the expression of adhesion molecule ligands on endothelial cells, sustaining the immune cell recruitment, antagonizing tumor-supportive immune cells like M2 macrophages, inducing tumor-microvasculature collapse by reducing tumor-supportive nutrients, sustaining the differentiation of antitumoral M1 macrophages, and inducing cancer cell apoptosis.^45,46^ TNF-α sustaining M1 polarization also promotes the release of antitumoral cytokines like IFN-γ, IL-8, and IL-2, which can amplify and sustain the antitumoral immune responses. More recently it has emerged that the dichotomy between M1 and M2 macrophages is reductive. In particular for the M2 macrophages, a more complex classification can be performed and it has emerged that M2 macrophages can be better identified as CD206+ cells, and that they can be better classified on the basis of different levels of expression. The differentiation based on CD206 expression is not just a simple phenotypic classification but also a functional differentiation (see ref (5)SI).

The ability of long-chain saturated fatty acids like palmitate and stearate to induce IL-8 upregulation in macrophages,^47−49^ as well as the possibility to use stearate- and palmitate-containing liposomes as adjuvants, is known and explored.^50^ It is worth noting that the adjuvant activity of stearate- and palmitate-containing liposomes is dependent on Mincle activation (proinflammatory activity).^51^ Conversely, Tn antigen interacts with the MGL receptor, whose activation enhances TLR2-mediated TNF-α production in macrophages.^52,53^ Since long-chain saturated fatty acid chains are TLR2 ligands able to induce proinflammatory responses, we can hypothesize that niosomes with their adjuvant ability can induce proinflammatory responses through Mincle or TLR2 activation and Tn (or Tn mimetic) antigen can modulate this response-binding MGL receptor, resulting in TNF-α production enhancement. Differently, in raw niosomes (no TnThr or TnThr-mimetic residues are displayed), the fatty acid-induced response predominates with a more prominent IL-8 production.

The recent development of nanocarriers based on polymeric, ceramic, and lipid biomaterials represents an efficient, organized delivery system for poorly active antigens in a more immunogenic shape. However, nano-sized carriers may have an intrinsic activity owing to their nature, directly activating host pathways and in some cases affecting cell viability. Therefore, we have evaluated the ability of niosomes-**2**, niosomes-**7**, and raw niosomes (blank) to interact with blood proteins and assessed their effects on the different cellular elements of blood. The adsorption of protein to nanomaterials is considered the main cause of the pathological host response to the biomaterial and so it represents an important aspect to be evaluated. Our results clearly suggested that the protein absorption on niosomes is negligible and consequently, also the pathological effect related to their use. Another important aspect to be considered is the possibility of niosomes to induce a toxic effect on the blood component. As is known, many amphiphilic molecules, including niosomes, when in contact with the red blood cells membrane induce its damage. Our results clearly showed that niosomes-**2**, niosomes-**7**, and raw niosomes did not have any impact on the integrity of the red blood cells membrane; indeed, no hemolysis was observed. In addition, the possible toxic effect of niosomes was screened on other cellular components of blood. In particular, we observed that neither glyco-niosomes nor raw niosomes affect the viability of white blood cells, indicating the absence of an undesired perturbation of immune cells and confirming their hemocompatibility.

All together, the biological results clearly show that glycosylated niosomes are able to induce the differentiation of macrophages toward a M1 phenotype with the potential to present the antigen to responder T lymphocytes in a proinflammatory microenvironment essential to induce an efficient antitumor immune response. Moreover, the ability of M1 macrophages to recruit and regulate other immune cells along with the ability to help in reverting the immunosuppressive environment is clearly suggested. It is worth noting that all of the niosomes studied resulted hemocompatible. These data open the way to further studies aimed at better exploring the use of glycosylated niosomes in *in vivo* models and their effects on lymphocyte activation and tumor growth.

## Conclusions

In conclusion, we reported on the synthesis of glycolipids **2** and **7** featuring, respectively, the TnThr antigen mimetic **1** and the native TnThr antigen as saccharide portions. Capitalizing on our experience in preparing glycosyl niosomes, glycolipids **2** and **7** were employed to assemble niosomes-**2** and niosomes-**7**, which were characterized and screened *in vitro*. An unpredictable difference between niosomes-**2** and niosomes-**7** was observed in terms of morphology (macroscopic level) and internal dynamics (microscopic level), which is very likely related to the different carbohydrate portions exposed on the niosomes’ surface. Although these observations deserve further studies, they confirm the role of sugar in “hardening” biomolecules.^54^ Niosomes-**2** and niosomes-**7** did not induce cell death, they were able to differentiate monocytes into M1 macrophages, as well as to induce the release of protective cytokines. In particular, niosomes-**2** induced a higher level of the CD11b marker and TNF-α release. This highlighted the potential of niosomes to present TACAs inducing *in vitro* an immune response without the presence of external adjuvants and confirms the role of TnThr mimetic **1** in protective immunostimulation. This approach well suits the development of synthetic tumor vaccines.

## Materials and Methods

### Synthesis of Acetylated Glycolipid **6**

To a solution of **4** (100 mg, 0.20 mmol) in dry CH_2_Cl_2_ (3 mL), HBTU (155 mg, 0.41 mmol), DIPEA (115 μL, 1.32 mmol), and hexadecylamine (75 mg, 0.36 mmol) were added, and the mixture was stirred at room temperature. After 1 h, the solvent was evaporated under reduced pressure to give the crude product **6**, as a pale-yellow oil, which was used without further purification.

### Synthesis of Glycolipid **7**

To a solution of crude **6** (0.40 mmol) in MeOH (1 mL), NH_3_ in MeOH 4 M (2 mL) was added. After stirring for 2 h, the reaction mixture was concentrated under vacuum and the residue was purified by flash chromatography (CH_2_Cl_2_/CH_3_OH 9:1) to give pure derivative **7** as a white foam (58 mg, 46% calculated over two steps). ^1^H NMR (500 MHz, DMSO) δ: 7.97–7.91 (m, 2H, 2 NH), 7.23 (d, *J*_NH-H2_ = 9.3 Hz, 1H, NH), 4.64–4.55 (m, 3H, H1, 2 OH), 4.36–4.25 (m, 2H, Hα, OH), 4.06–3.96 (m, 2H, Hβ, H2), 3.72 (m, 1H, H4), 3.70–3.58 (m, 2H, H5, H3), 3.55–3.47 (m, 1H, H6a), 3.47–3.40 (m, 1H, 6b), 3.17–3.08 (m, 1H, H1′), 2.97–2.87 (m, 1H, H1′), 1.95, 1.88 (s, 6H, Ac), 1.42–1.33 (m, 2H, CH_2_), 1.24 (s, 26H, 14 CH_2_), 1.10 (d, *J*_CH3-Hβ_ = 6.4 Hz, 3H, CH_3_Thr), 0.86 (t, *J* = 7.1 Hz, 3H, H2′).

^13^C NMR (125 MHz, DMSO) δ: 170.3, 170.2, 170.1 (CONH), 99.9 (CH, C1), 75.7 (CH, Cβ), 72.1 (CH, C5), 68.7 (CH, C3), 68.6 (CH, C4), 61.0 (CH_2_, C6), 57.0 (CH, Cα), 50.0 (CH, C2), 39.2 (CH_2_, C1′), 29.5, 29.5, 29.4, 29.2, 26.9 (CH_2_), 23.4, 23.1 (CH_3_, Ac), 22.6 (CH_2_), 18.9 (CH_3_, CH_3_Thr), 14.4 (CH_3_, C2′).

### Synthesis of Acetylated Glycolipid **5**

To a solution of **3** (200 mg, 0.40 mmol) in dry CH_2_Cl_2_ (6 mL), HBTU (330 mg, 0.87 mmol), DIPEA (230 μL, 2.6 mmol), and hexadecylamine (150 mg, 0.72 mmol) were added, and the mixture was stirred at room temperature. After 1 h, the solvent was evaporated under reduced pressure to give the crude product **5**, as a pale-yellow oil, which was used without further purification.

### Synthesis of Glycolipid **2**

To a solution of crude **5** (0.20 mmol) in MeOH (1 mL), NH_3_ in MeOH 4 M (2 mL) was added. After stirring for 2 h, the reaction mixture was concentrated under vacuum and the residue was purified by flash chromatography (CH_2_Cl_2_/CH_3_OH 9:1) to give pure derivative **2** as a white solid (151 mg, 62% calculated over two steps). ^1^H NMR (500 MHz, DMSO) δ: 7.88 (t, *J*_NH-H1′_ = 5.6 Hz, 1H, NH), 7.36 (d, *J*_NH-Hα_ = 2.1 Hz, 1H, NH), 5.55 (d, *J*_H1-H2_ = 2.6 Hz, 1H, H1), 5.17 (d, *J*_OH-H3_ = 6.7 Hz, 1H, OH), 4.81 (d, *J*_OH-H4_ = 4.6 Hz, 1H, OH), 4.69 (t, *J*_OH-H6a,H6b_ = 5.5 Hz, 1H, OH), 4.02 (m, 1H, Hα), 3.85 (m, 1H, H5), 3.76 (m, 1H, H4), 3.60–3.54 (m, 1H, H6a), 3.53–3.44 (m, 2H, H6b, H3), 3.30 (m, 1H, H2), 3.12–3.00 (m, 2H, H1′), 2.78 (m, 1H, Hβa), 2.57 (m, 1H, Hβb), 1.45–1.34 (m, 2H, CH_2_), (s, 26H, CH_2_), 0.86 (t, *J*_H22-H21_ = 6.7 Hz, H2′).

^13^C NMR (125 MHz, DMSO) δ: 170.2, 164.9 (CONH), 155.0 (Cq), 96.6 (CH, C1), 96.3 (Cq), 74.2 (CH, C5), 68.9 (CH, C4), 65.8 (CH, C3), 60.8 (CH_2_, C6), 52.1 (CH, Cα), 39.3 (CH, C2), 39.2 (CH_2_, C1′), 31.8 (CH_2_), 31.2 (CH2, Cβ), 29.5, 29.5, 29.4 (CH_2_), 29.2, 29.2, 26.8, 22.6 (CH_2_), 14.4 (CH_3_, C2′).

## Supporting Information Available

Material and methods, NMR spectra of compounds **2**, **5**–**7**, synthesis and characterization of niosomes, niosomes stability (PDF)
