# Supplementary material for: Niosomes as Biocompatible Scaffolds for the Multivalent Presentation of Tumor-Associated Antigens (TACAs) to the Immune System
Source: Bioconjug Chem. 2022 Dec 15;34(1):181–92. doi: 10.1021/acs.bioconjchem.2c00383 (PMC9853506; doi:10.1021/acs.bioconjchem.2c00383)
Supplement: Supplementary file 1 — bc2c00383_si_001.pdf [file bc2c00383_si_001.pdf]

Supporting Information

Niosomes as Biocompatible Scaffolds for the Multivalent Presentation of Tumor Associated Antigens (TACAs) to Immune System

Silvia Fallarini,[a] Francesco Papi,[b] Federico Licciardi,[b] Francesca Natali,[c] Grazia Lombardi,[a] Francesca Maestrelli[b,*], Cristina Nativi[b,*]

[a] Department of Pharmaceutical Sciences, University of "Piemonte Orientale", Novara 28100, Italy; [b] Department of Chemistry, University of Florence, Sesto Fiorentino (FI) 50019, Italy; [c] CNR-IOM and INSIDE@ILL, c/o OGG, 71 avenue des Martyrs, 38042 Grenoble Cedex 9, France.

*Corresponding authors: cristina.nativi@unifi.it; francesca.maestrelli@unifi.it

Table of Content

Material and Methods	Pag.	S2
Synthesis of acetylated glycolipid **6**		S2
Synthesis of glycolipid **7**		S2
Synthesis of acetylated glycolipid **5**		S3
Synthesis of glycolipid **2**		S3
Data S1**:** ^{1}H, ^{13}C, 2D NMR spectra of glycolipid **2**		S5
Data S2: ^{1}H, ^{13}C, 2D NMR spectra of glycolipid **7**		S8
Preparation of niosomes		S10
Niosomes characterization and stability studies		S10
Table S1		S11
Niosomes internal dynamics		S11
Figure S1		S12
Figure S2		S12
In vitro tests		S13
Figure S3		S14
Figure S4		S15
Figure S5		S16
Figure S6		S17
References		S17

Materials and methods

Analytical grade solvents and commercially available reagents were used without further purification. Silica gel flash column chromatography purifications were performed using Geduran® Si 60 (0.040-0.063 mm). TLC analyses were performed on glass Merck silica gel 60 F_{254} plates. ^{1}Hydrogen, ^{13}Carbon and Two-Dimensional Nuclear Magnetic (^{1}H NMR, ^{13}C NMR and 2D-NMR) spectra were recorded on a 500 MHz Bruker AVANCE II at 298 K. The assignments of resonances, including those for compounds mixture, were made on the basis of cross correlation observed in 2D NMR experiments. Multiplicity abbreviations: b = broad, s = singlet, d = doublet, t = triplet, q = quartet, and m =multiplet, were used.

Synthesis of acetylated glycolipid 6

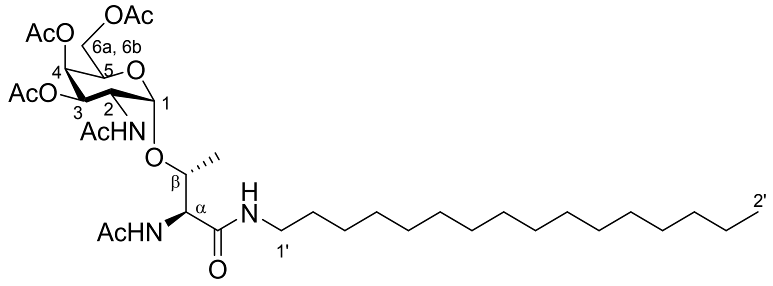

To a solution of **4** (100 mg, 0.20 mmol) in dry CH_2Cl_2 (3 mL), HBTU (155 mg, 0.41 mmol) DIPEA (115 μL, 1.32 mmol) and hexadecylamine (75 mg, 0.36 mmol) were added, and the mixture was stirred at room temperature. After 1 h, the solvent was evaporated under reduced pressure, to give the crude product **6**, as a pale-yellow oil, which was used without further purification.

Synthesis of glycolipid 7

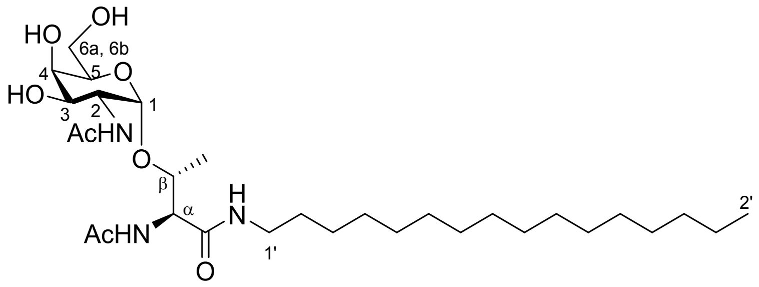

To a solution of crude **6** (0.40 mmol) in MeOH (1 mL), NH_3 in MeOH 4M (2 mL) was added. After stirring for 2 h, the reaction mixture was concentrated under vacuum and the residue was purified by flash chromatography (CH_2Cl_2/CH_3OH 9:1) to give pure derivative **7** as a white foam (58 mg, 46% calculated over two steps).

^{1}**H NMR** (500 MHz, DMSO) δ: 7.97-7.91 (m, 2H, 2 NH), 7.23 (d, $J_{NH\text{-}H2}$ = 9.3 Hz, 1H, NH), 4.64-4.55 (m, 3H, H1, 2 OH), 4.36-4.25 (m, 2H, Hα, OH), 4.06-3.96 (m, 2H, Hβ, H2), 3.72 (m, 1H, H4), 3.70-3.58 (m, 2H, H5, H3), 3.55-3.47 (m, 1H, H6a), 3.47-3.40 (m, 1H, 6b), 3.17-3.08 (m, 1H, H1'), 2.97-2.87 (m, 1H, H1'), 1.95, 1.88 (s, 6H, Ac), 1.42-1.33 (m, 2H, CH_2), 1.24 (s, 26H, 14 CH_2), 1.10 (d, $J_{CH3\text{-}H\beta}$ = 6.4 Hz, 3H, CH_3Thr), 0.86 (t,= 7.1 Hz, 3H, H2').
^{13}**C NMR** (125 MHz, DMSO) δ: 170.3, 170.2, 170.1 (CONH), 99.9 (CH, C1), 75.7 (CH, Cβ), 72.1 (CH, C5), 68.7 (CH, C3), 68.6 (CH, C4), 61.0 (CH_2, C6), 57.0 (CH, Cα), 50.0 (CH, C2), 39.2 (CH_2, C1'), 29.5, 29.5, 29.4, 29.2, 26.9 (CH_2), 23.4, 23.1 (CH_3, Ac), 22.6 (CH_2), 18.9 (CH_3, CH_3Thr), 14.4 (CH_3, C2').

Synthesis of acetylated glycolipid 5

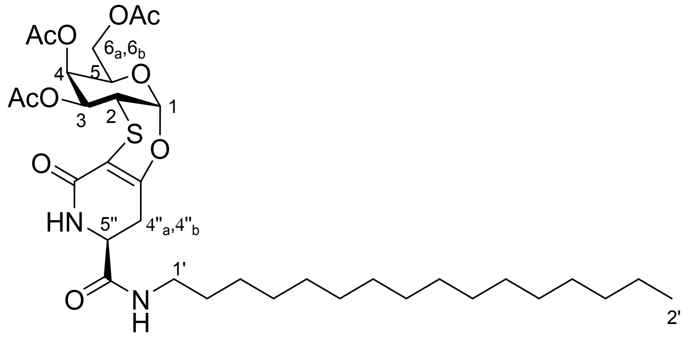

To a solution of **3** (200 mg, 0.40 mmol) in dry CH_2Cl_2 (6 mL), HBTU (330 mg, 0.87 mmol) DIPEA (230 µL, 2.6 mmol) and hexadecylamine (150 mg, 0.72 mmol) were added, and the mixture was stirred at room temperature. After 1 h, the solvent was evaporated under reduced pressure, to give the crude product **5**, as a pale-yellow oil, which was used without further purification.

Synthesis of glycolipid 2

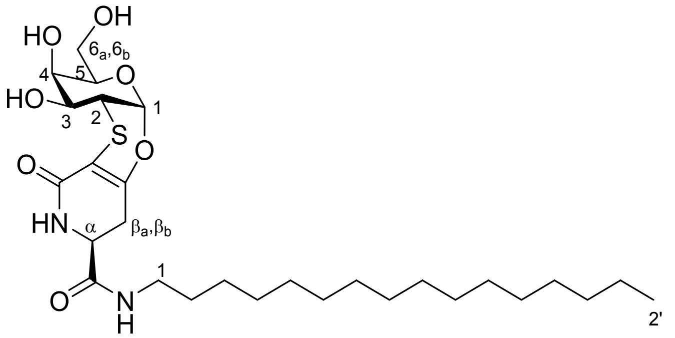

To a solution of crude **5** (0.20 mmol) in MeOH (1 mL), NH_3 in MeOH 4M (2 mL) was added. After stirring for 2 h, the reaction mixture was concentrated under vacuum and the residue was purified by flash chromatography (CH_2Cl_2/CH_3OH 9:1) to give pure derivative **2** as a white solid (151 mg, 62% calculated over two steps).

^{1}**H NMR** (500 MHz, DMSO) δ: 7.88 (t, $J_{NH\text{-}H1'}$ = 5.6 Hz, 1H, NH), 7.36 (d, $J_{NH\text{-}H\alpha}$ = 2.1 Hz, 1H, NH), 5.55 (d, $J_{H1\text{-}H2}$ = 2.6 Hz, 1H, H1), 5.17 (d, $J_{OH\text{-}H3}$ = 6.7 Hz, 1H, OH), 4.81 (d, $J_{OH\text{-}H4}$ = 4.6 Hz, 1H, OH), 4.69 (t, $J_{OH\text{-}H6a,H6b}$ = 5.5 Hz, 1H, OH), 4.02 (m, 1H, Hα), 3.85 (m, 1H, H5), 3.76 (m, 1H, H4), 3.60-3.54 (m, 1H, H6a), 3.53-3.44 (m, 2H, H6b, H3), 3.30 (m, 1H, H2), 3.12-3.00 (m, 2H, H1'), 2.78 (m, 1H, Hβa), 2.57 (m, 1H, Hβb), 1.45-1.34 (m, 2H, CH_2), (s, 26H, CH_2), 0.86 (t, $J_{H22\text{-}H21}$ = 6.7 Hz, H2').

^{13}**C NMR** (125 MHz, DMSO) δ: 170.2, 164.9 (CONH), 155.0 (Cq), 96.6 (CH, C1), 96.3 (Cq), 74.2 (CH, C5), 68.9 (CH, C4), 65.8 (CH, C3), 60.8 (CH_2, C6), 52.1 (CH, Cα), 39.3 (CH, C2), 39.2 (CH_2, C1'), 31.8 (CH_2), 31.2 (CH_2, Cβ), 29.5, 29.5, 29.4 (CH_2) , 29.2, 29.2, 26.8, 22.6 (CH_2), 14.4 (CH_3, C2').

Data S1: ^{1}H, ^{13}C, 2D NMR spectra of glycolipid **2** in DMSO(d6)

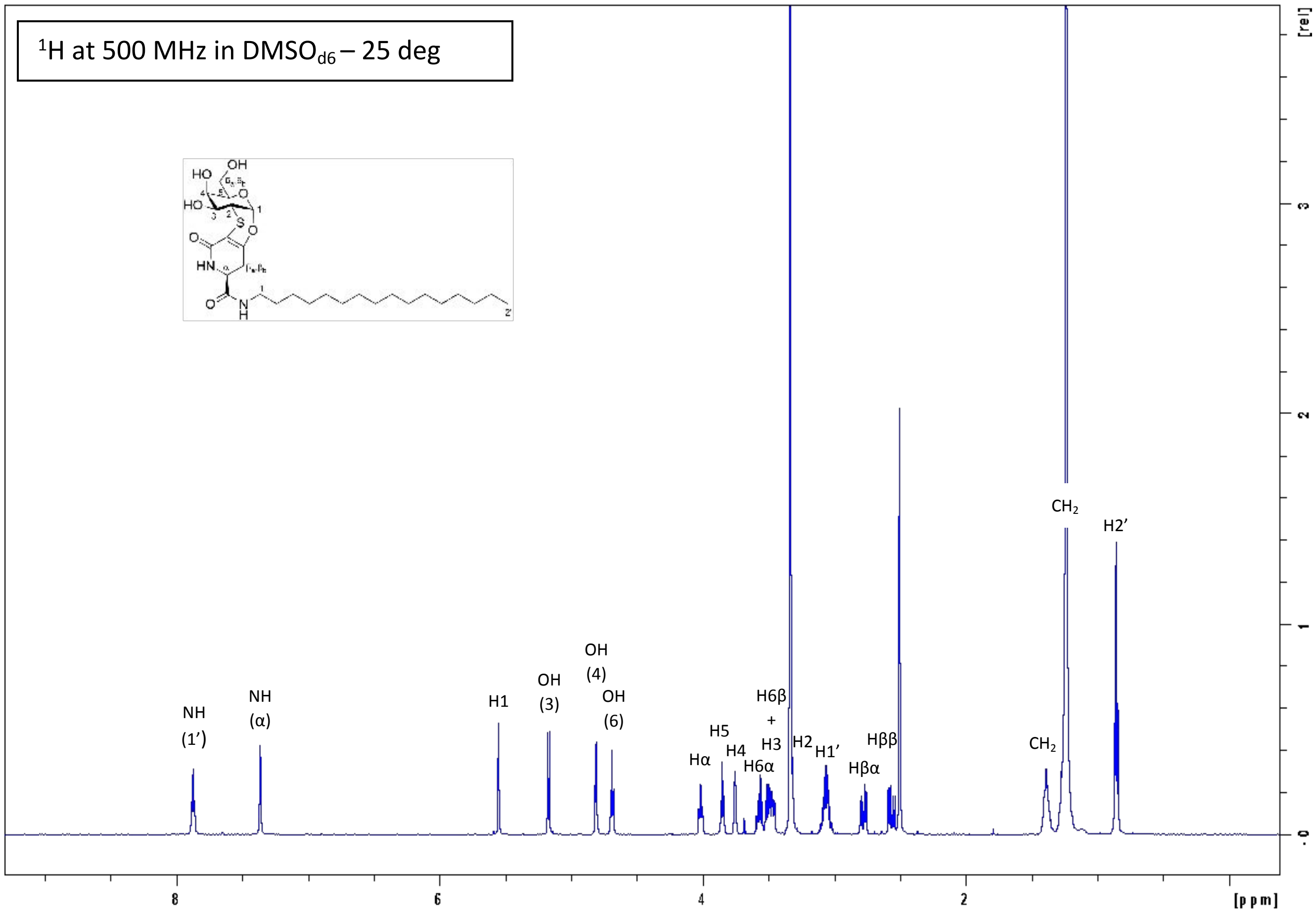

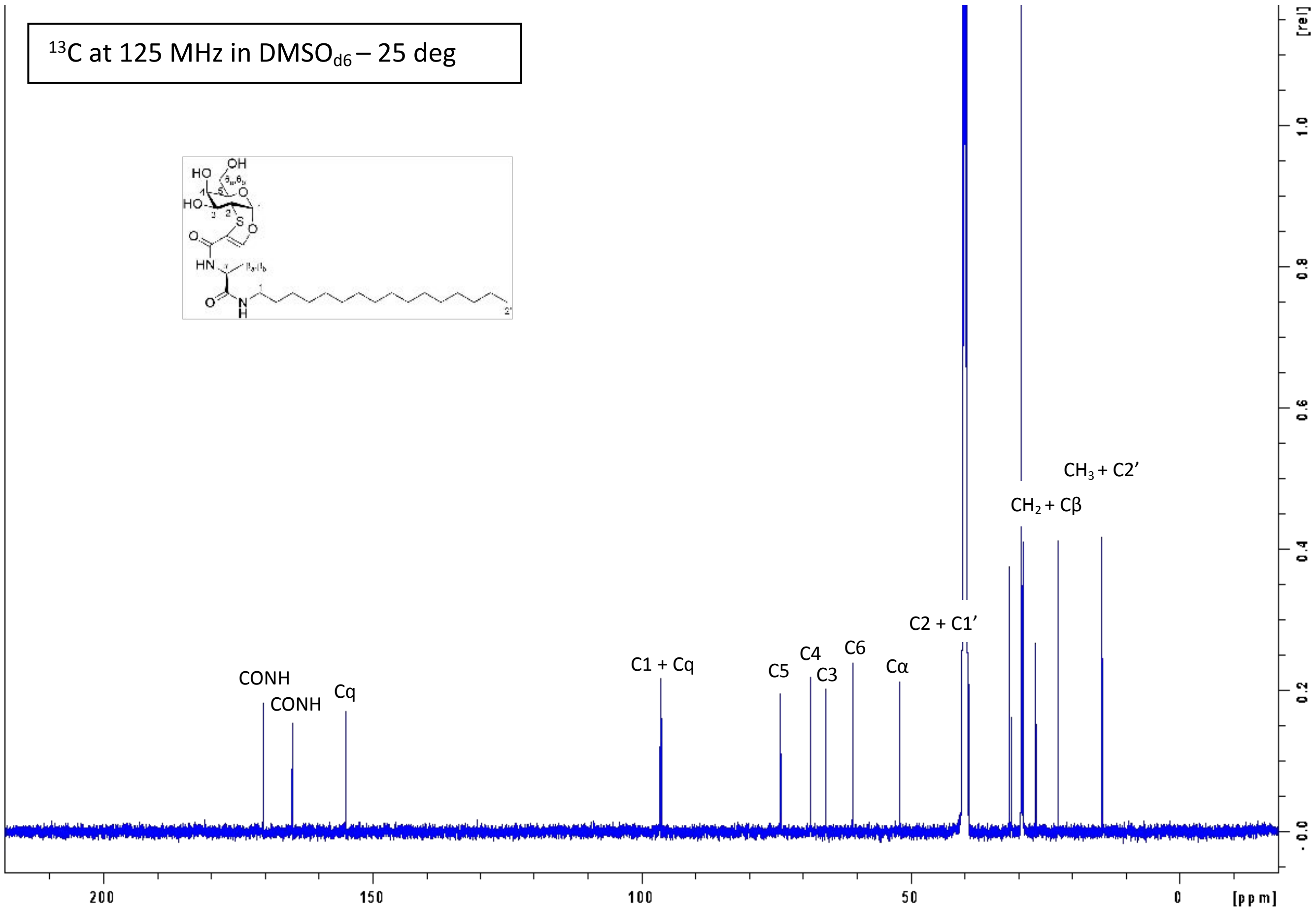

^{13}C at 125 MHz in $DMSO_{d6}$ – 25 deg
CONH
CONH
Cq
C1 + Cq
C5
C4
C3
C6
Cα
C2 + C1’
CH_2 + Cβ
CH_3 + C2’
[rel]
[ppm]

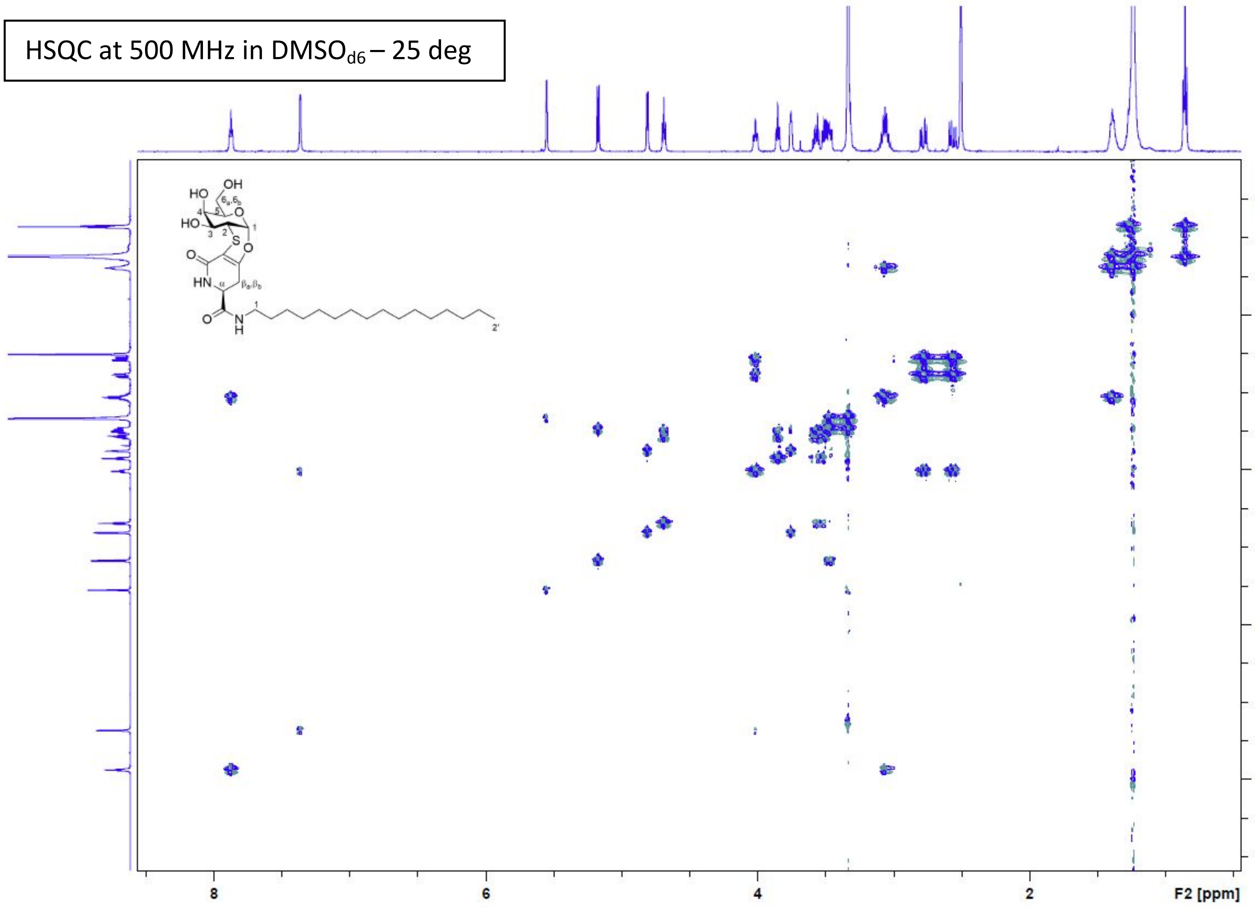

HSQC at 500 MHz in $DMSO_{d6}$ – 25 deg
F1 [ppm]
F2 [ppm]

COSY at 500 MHz in $DMSO_{d6}$ – 25 deg

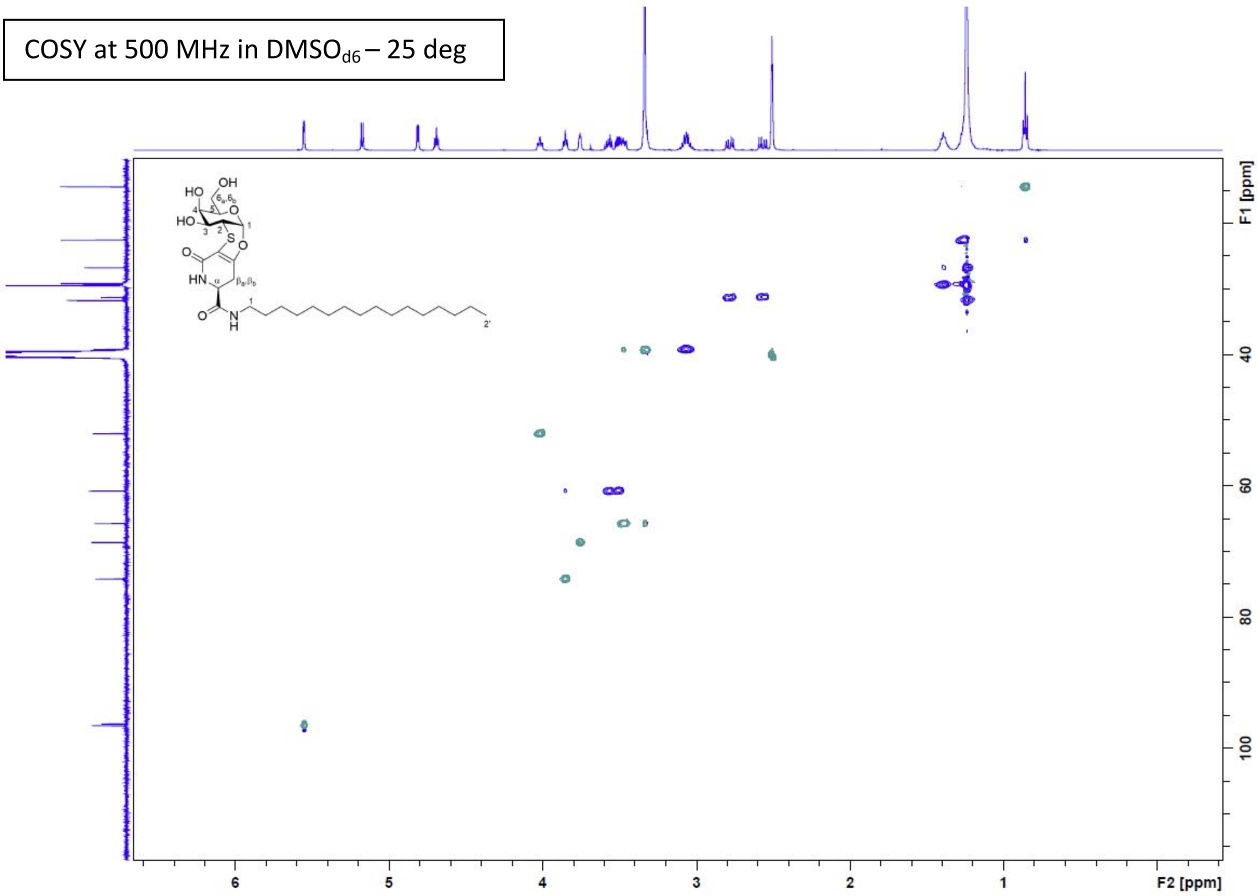

Data S2: ^{1}H, ^{13}C, 2D NMR spectra of glycolipid **7** in DMSO(d6)

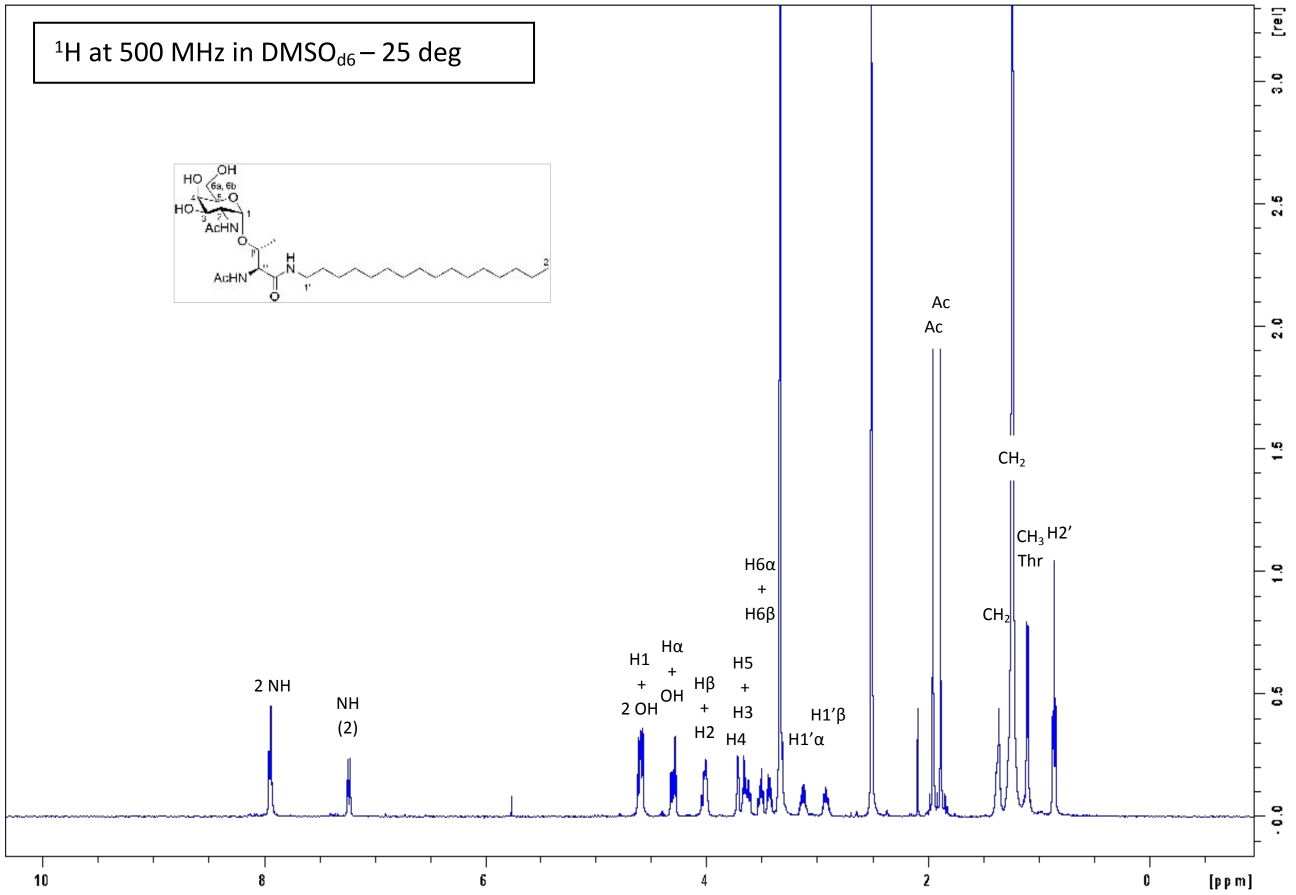

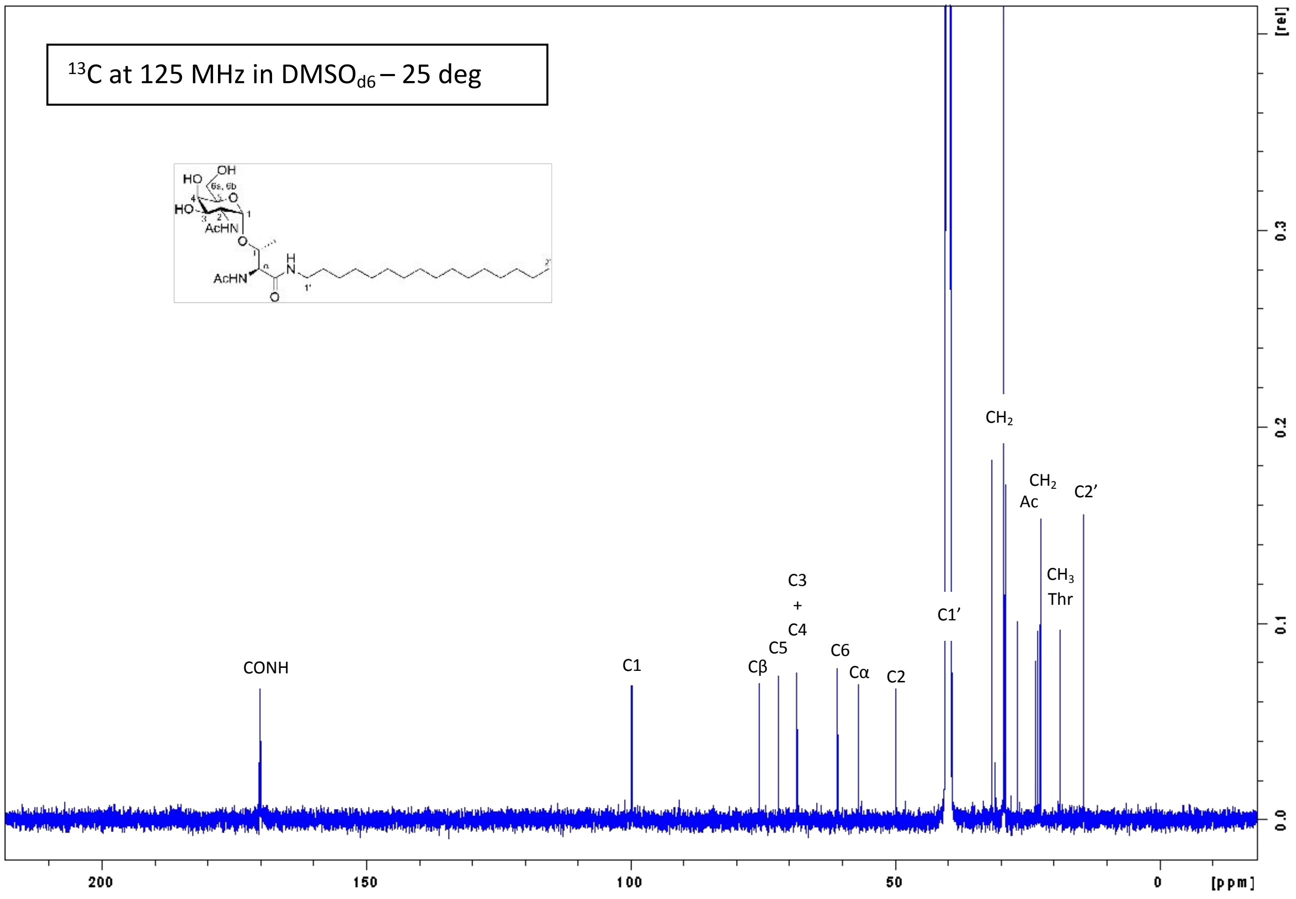

HSQC at 500 MHz in $DMSO_{d6}$ – 25 deg

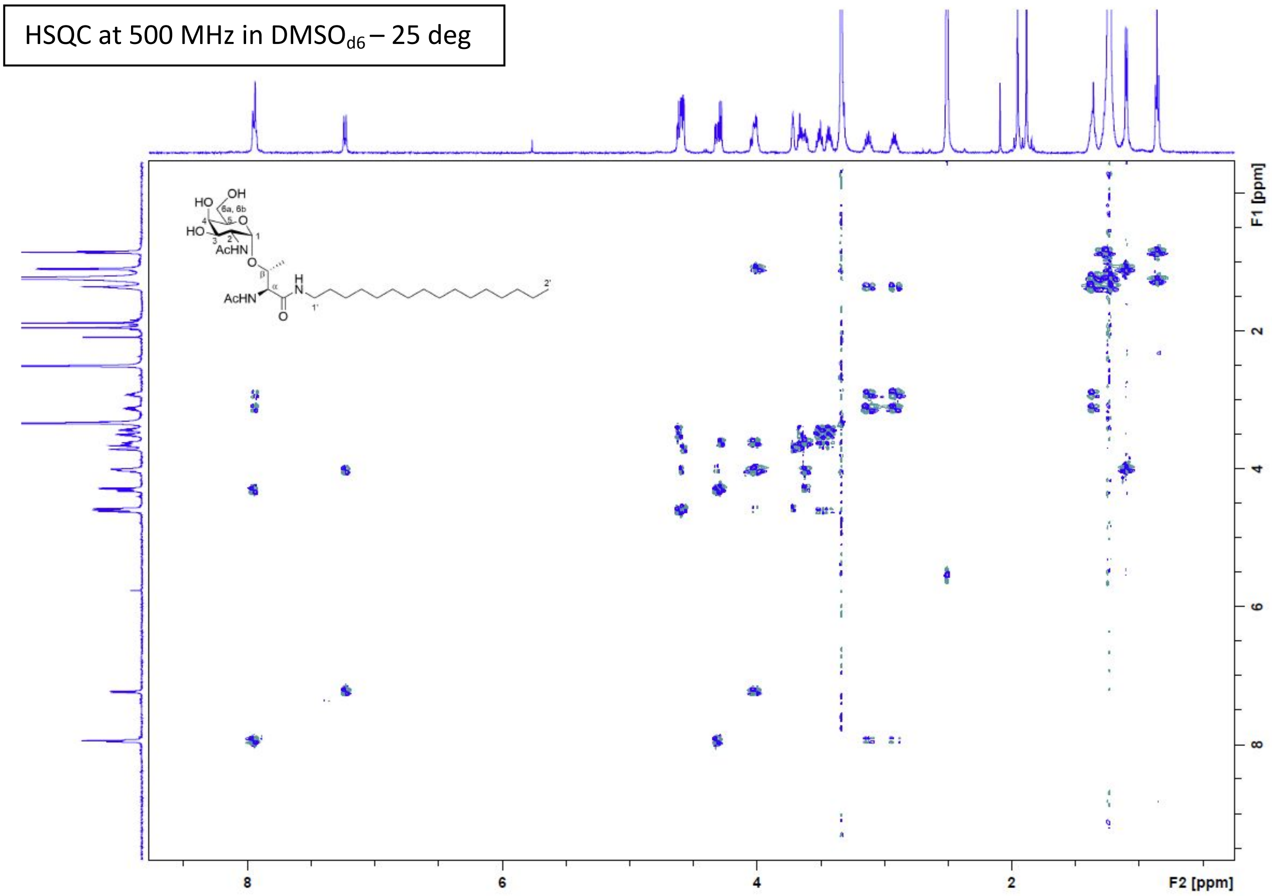

COSY at 500 MHz in $DMSO_{d6}$ – 25 deg

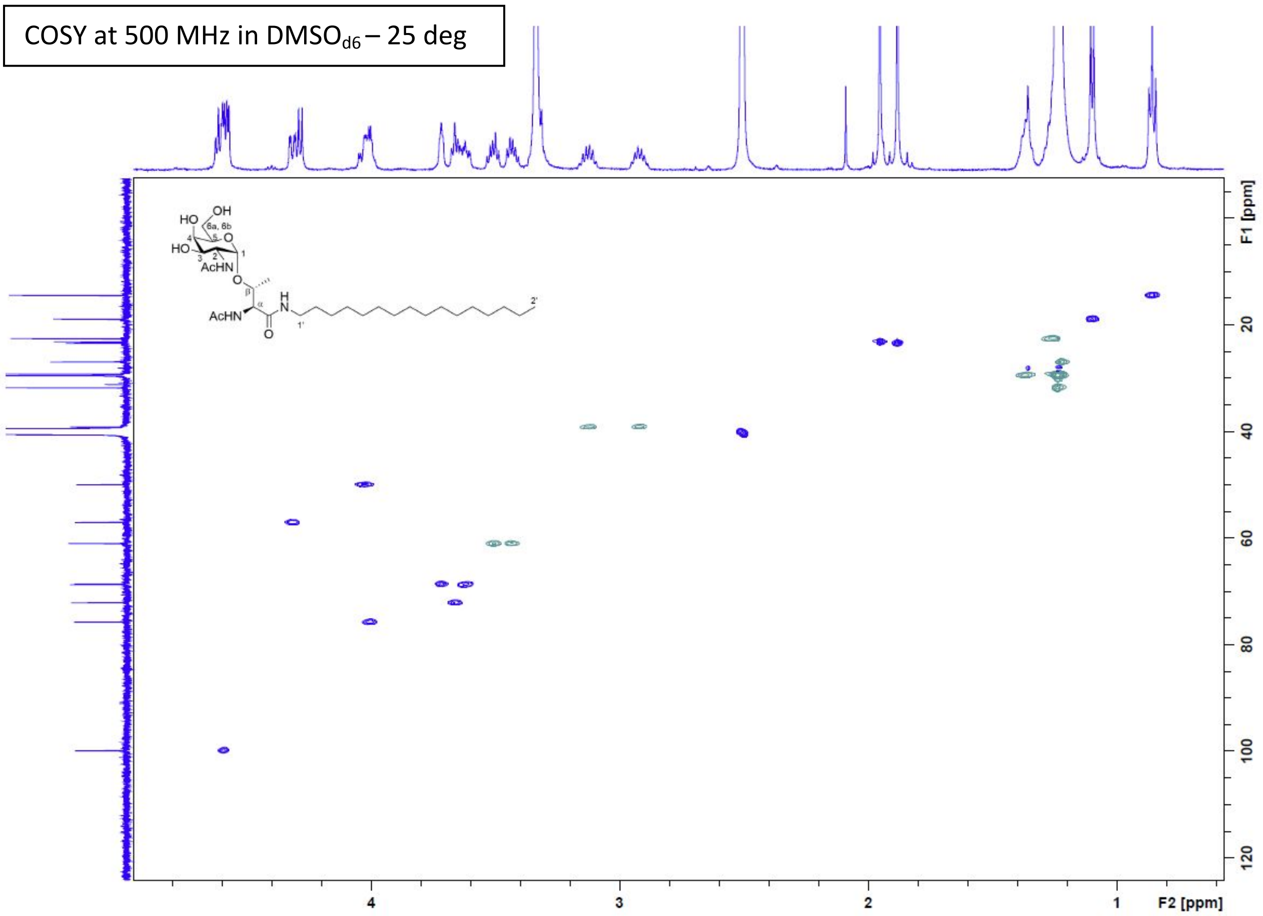

Preparation of niosomes

Solulan™ C24 (SOL) (Poly-24-oxyethylenecholesteryl ether) was a gift by Lubrizol (Cleveland, Ohio, USA). Cholesterol (CH), sorbitan stearate (Span 60, HLB 4.7), were purchased from Merck Life Science S.r.l. (Milan, Italy). All the other reagents were of analytical grade.

Niosomes were prepared by thin layer evaporation-paddle that is a partial modification of a previous method.[1,2] Briefly, the lipidic phase, composed by Span 60/CH/SOL/glycolipid **2** or **7** in a 4.0:2.5:2.5:1 w/w proportion was dissolved in a $CHCl_3$/CH_3OH 1:1 mixture and solvents were completely removed by rotary evaporation under vacuum to form a thin layer on the flask wall. Under N_2 atmosphere, the layer was hydrated with 20 mL of D_2O under stirring by a paddle at 2000 rpm for 30 minutes, heating in a water bath at 65°C. Raw niosomes (without glycolipid **2** or glycolipid **7**) were prepared as control (blank). The total amount of lipid components was 19.06 mg/mL. All the suspensions were centrifuged at 4000 rpm for 15 minutes with a Z 200 A centrifuge (Hermle LaborTechnik GmbH, Wehingen, Germany) according to a previous method.[3] The supernatant was collected and subjected to sonication in an ice bath with a Sonoplus HD2200 ultrasonic homogenizer (Bandelin Electronic GmbH, Berlin, Germany) equipped with KE76 probe at 50% of power for 5 minutes for 2 cycles. After preparation, niosomes were lyophilized with a Lio 5Pascal machine (Milan, Italy). Before use lyophilized niosomes were reconstituted with 20 mL of D_2O and subjected to sonication in an ice bath with a Sonopuls HD2200 ultrasonic homogenizer (Bandelin Electronic GmbH, Berlin, Germany) equipped with KE76 probe at 50% of power for 5 minutes for 5 cycles.

Niosomes characterization and stability studies

Particle size, polydispersion index (PDI) and ζ-potential of niosomes were measured by dynamic light scattering using a Zetasizer Nanoseries ZS90 (Malvern Instrument, Worcestershire, UK) at an angle of 90 in 0.01m width cells at 25±1°C. Colloidal suspensions were properly diluted with distilled water in order to avoid scattering phenomena. Each sample was analyzed in triplicate. The morphology was evaluated using a Scanning transmission electronic (STEM) Microscope (FIB-SEM Gaia 3,Tescan s.r.o, Brno, Czech Republic). The samples were stained with 2% w/v phosphotungstic acid solution and placed on copper grids with carbon films for viewing. Stability studies were conducted during 8 hours at 37°C by diluting the niosomal suspension 1:8 v/v with Hanks′ Balanced Salt solution (HBSS) pH 7. 4. The samples were placed in vials and analyzed each hour in terms of mean particle size, PDI and ζ-potential as previously described.

Neutron scattering experiments.

Neutron scattering measurements were performed on the thermal (λ = 2.23Å) high-energy resolution backscattering spectrometer IN13 (Institut Laue-Langevin, Grenoble, France) which is characterized by a very large momentum transfer range ($0.25 < Q < 4.9$ Å^{-1}) with a good and nearly Q-independent energy resolution (8 μeV FWHM). Therefore, IN13 allows accessing the space and time windows of 1 - 25 Å and 0.1 ns respectively.The neutron beam scattered from the sample was reflected in almost perfect backscattering geometry by CaF_2 analysers to be finally collected by 35 He^3 detectors in the region $1.1 < Q < 4.9$ Å^{-1} and by a Position Sensitive He^3 Detector (PSD) in the low Q region ($0.3 < Q < 0.8$ Å^{-1}). The elastic energy value ($\omega = 0$) was kept fixed within 3 μeV of accepted tolerance. Elastic Incoherent Neutron Scattering was recorded using niosomes prepared (blank, niosome-**2** and niosome-**7**). Niosomes were prepared in D_2O as a buffer to avoid the contribution of water hydrogens during EINS analysis. Elastic scans (ENS) were measured as function of the temperature (278-318 K). The elastic scattering intensities ($I_{el}(Q) \equiv S(Q, \omega=0)$), suitably corrected for the empty sample holder contribution, were normalised with respect to a vanadium scan to compensate for spurious background contributions and detector efficiency. Samples concentrated at 40 mg/mL (2 mL) were

exposed to the neutron beam in order: 1) to be sufficiently high to gain in signal-to-noise ratio and 2) not too high to minimize the neutron absorption from the sample, thus avoiding correction from multiple scattering contribution.

Table S1. Freshly prepared and after reconstitution niosomes' characterization

	Freshly prepared			After reconstitution		
**Batch**	**Particle size (nm)**	**PDI**	**ζ-potential (mV)**	**Particle size (nm)**	**PDI**	**ζ-potential (mV)**
Blank	214.3 ± 3.4	0.412	-51.8 ± 1.4	139.5 ± 3.6	0.377	-42.6 ± 1.2
Niosomes-**7**	194.4 ± 2.6	0.274	-26.6 ± 0.9	174.2 ± 2.4	0.368	-20.3 ± 2.4
Niosomes-**2**	175.8 ± 2.8	0.247	-38.9 ± 1.8	133.9 ± 3.7	0.275	-39.2 ± 1.4

Niosome internal dynamics

Neutron scattering measurements were performed on the thermal (λ = 2.23Å) high-energy resolution backscattering spectrometer IN13 (Institut Laue-Langevin, Grenoble, France) which is characterized by a very large momentum transfer range ($0.25 < Q < 4.9$ Å^{-1}) with a good and nearly Q-independent energy resolution (8 μeV FWHM). Therefore, IN13 allows accessing the space and time windows of 1 - 25 Å and 0.1 ns respectively.The neutron beam scattered from the sample was reflected in almost perfect backscattering geometry by CaF_2 analysers to be finally collected by 35 He^3 detectors in the region $1.1 < Q < 4.9$ Å^{-1} and by a Position Sensitive He^3 Detector (PSD) in the low Q region ($0.3 < Q < 0.8$ Å^{-1}). The elastic energy value ($\omega = 0$) was kept fixed within 3 μeV of accepted tolerance. Elastic Incoherent Neutron Scattering was recorded using niosomes prepared (blank, niosome-**2** and niosome-**7**). Niosomes were prepared in D_2O as a buffer to avoid the contribution of water hydrogens during EINS analysis. Elastic scans (ENS) were measured as function of the temperature (278-318 K). The elastic scattering intensities ($I_{el}(Q) \equiv S(Q, \omega=0)$), suitably corrected for the empty sample holder contribution, were normalised with respect to a vanadium scan to compensate for spurious background contributions and detector efficiency. Samples concentrated at 40 mg/mL (2 mL) were exposed to the neutron beam in order: 1) to be sufficiently high to gain in signal-to-noise ratio and 2) not too high to minimize the neutron absorption from the sample, thus avoiding correction from multiple scattering contribution.

The experimental data consist of the elastic neutron scattering intensity $S(Q, \omega = 0)$ vs. the squared momentum transfer. In the low Q region, the Q-dependence of $S(Q, \omega = 0)$ is given, according to the Gaussian approximation, by equation S1:

$$S(Q,=0)=I_0\exp^{-\frac{<u^2>Q^2}{6}} \qquad (S1)$$

where I_0 is a constant and $<u^2>$ is the mean-square amplitude of atomic displacements (MSD). The elastic neutron intensity is reported in Figure S1 for the niosome-**2**, as example.

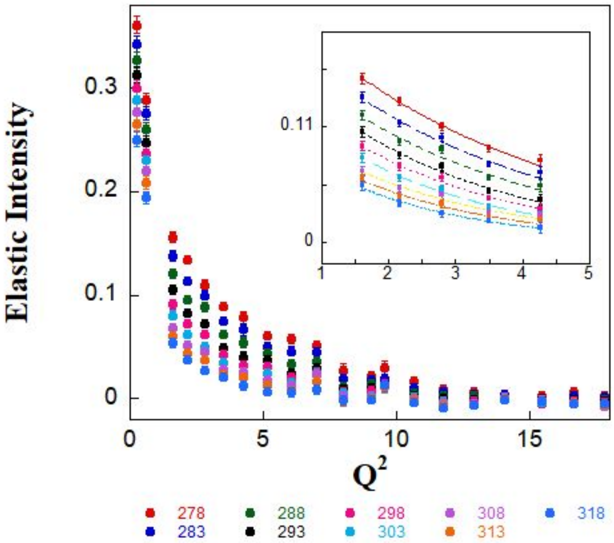

Figure S1: Temperature behavior of the elastic intensities of niosome-**2** sample

Temperatures lower than 278 K were not investigated to avoid data contamination from coherent contribution arising from ice Bragg peaks. The typical temperature dependence is observed, in which the signal decreases with increasing T due to the acquired proton dynamics. Indeed, the elastic scattering provides an insight of vibrational motion of the protons in the sample. Due to the large incoherent cross section of H atoms, with respect to N, C, O etc., the technique is particularly sensitive to the H dynamics. Using heavy water allows us to neglect the scattering contribution from the buffer (being 2:80, D:H, the corresponding ratio of incoherent cross section). As expected for a typical solution, the elastic intensity decreases rapidly in Q. Thus, from the slope of the semi-logarithmic plot of the incoherent scattering function, the Mean Square Displacements (MSD) for a given T are extracted. MSD are plotted in Figure S2 (left) for all the samples.

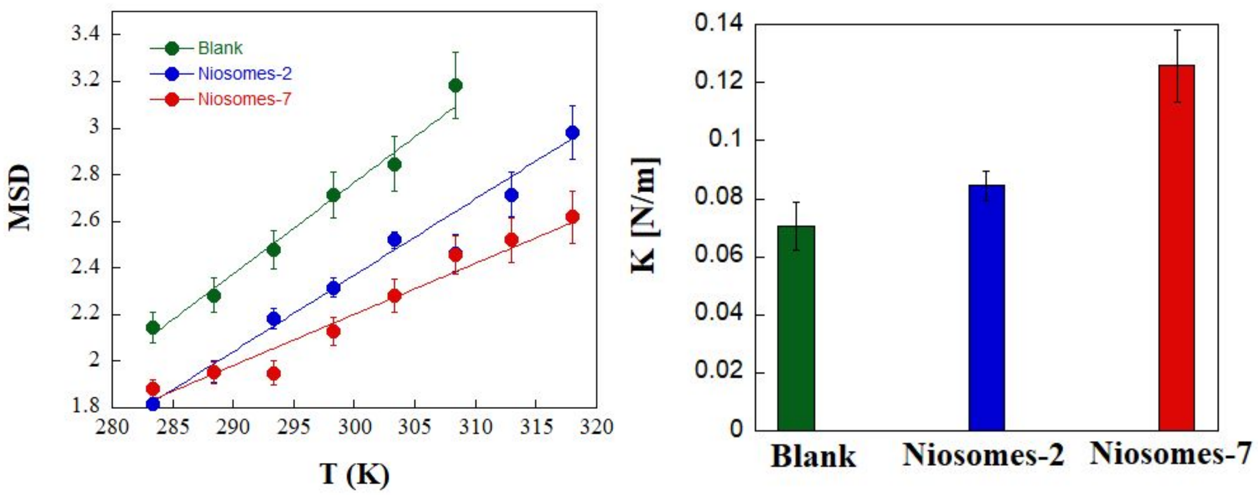

Figure S2: Left: MSD Temperature behavior of the elastic intensities. Lines are fitted using eq. 2. Right: Histograms of K values extracted from the MSDs.

The temperature variation of the MSDs can now be interpreted in terms of an empirical effective force constant called , called resilience by Zaccai G.[4] It describes the rigidity or resilience of the proteins or macromolecules. The effective average force constant for sample dynamics, < k >, can then be calculated from the slope of MSD ($<u^2>$) as a function of temperature, by applying a quasi-harmonic approximation (equation S2:

$$<k'>=\frac{0.00276}{\frac{d<u^2>}{dT}} \qquad \text{(S2)}$$

K values for blank niosomes and under TnThr natural/mimetic addition, are compared in Figure S2 (right).

In vitro tests

THP1 and U937 cell culture and differentiation. THP1 and U937 cell lines were obtained from ATCC and maintained in RPMI 1640 medium supplemented with 10% of fetal bovine serum (FBS), 2 mmol/L L-glutammine (Immunological Sciences, Rome, Italy) and 1 mg/mL kanamycin (Sigma-Aldrich Milan, Italy), used to prevent bacterial contamination of cell cultures. THP1 cells (2 x 10^5 cells/mL) and U937 cells (8 x 10^5 cells/mL) were seeded in 6 multiwell and differentiated into macrophages (M0) by 24 h incubation with 150 or 100 nM phorbol 12-mystrate-13-acetate (PMA) respectively (Sigma-Aldrich) followed by 24 h incubation in RMPI medium.

M0 were polarized into M1 macrophages by incubation with 0.5 µg/mL lipopolysaccharide (LPS) (Sigma Aldrich, Milan Italy) or 20 ng/mL of IFN-γ (Promega, Milan Italy). Differentiated macrophages (M0) were untreated/treated with increasing concentrations (0.1 – 10 µg/mL) of tested compounds.

Flow cytometry. Flow cytometry analyses were performed using the Attune NxT (Thermo Fisher Scientific, Italy). Forward (FCS) and side (SSC) scatters were used to identify cell populations and measure size and granularity of the cells. Auto-fluorescence was recorded by analysing unstained cells in the FL-1 channel (blue laser; excitation 488, emission 530/30). For detection of cell surface

markers 1 μg/mL of monoclonal mouse anti-human antibodies CD14-FITC, CD86-PE, CD11b-PeCy7 were used for each sample. After 24h incubation, untreated/treated cells were harvested, washed, incubated with the antibodies for 30 minutes on ice in the dark. Labelled samples were washed, resuspended in PBS and analyzed by FACS, for each sample 10000 events were recorded. All data were analyzed using FCS express 7 (Flow cytometry software, DeNovo software).

Cell viability. After 24 h incubation, untreated/treated cells were harvested, washed, incubated with 2 μg/mL of propidium iodide (PI) for 30 min on ice in the dark and kept ice-cold until FACS analysis.

ELISA tests. After 48 h incubation, culture media of untreated/treated cells were collected and stored in –80 °C until analysis. Levels of IL-8, TNF-α and IL-10 were measured by ELISA assay according to manufacturer's guidelines (Biolegend® San Diego, CA, USA).

Hemocompatibility assays

Niosomes hemocompatibility was tested using freshly isolated peripheral blood obtained by healthy volunteer after their informed consent.

Protein concentration. Protein concentration levels in donors' blood samples treated with Niosomes were measured using an indirect method to assess the early interaction between Niosomes and blood components. Samples (200 μL) of peripheral blood was dispensed to the wells of 96-well tissue culture plates and treated with increasing concentrations (0.01-100 μg/mL) of tested compounds in triplicate. Samples were incubated in a 37°C, 5%CO_2 humidified incubator under static condition. After 24h plates were centrifuged at 700 g for 10 min, 100 μL of supernatants were collected and immediately tested for protein concentration. Protein concentrations were measured by Bradford assay (Fermentas) on 5 μL of blood supernatant and data expressed as μg/mL calculated by a bovine serum albumin standard curve.

Blood hemolysis test. Blood was centrifuged at 700 g for 10 min to separate the red blood cells (RBCs) from the plasma. The isolated RBCs were washed three times with 150 mM NaCl solution and centrifuged again as above. The RBC pellet was suspended in 20mL of PBS. The RBC suspension (200 μL), was added to test samples (800 μL, final volume; 0.1–100 μg/mL test compound concentrations). Triton-X 100 (1%) was used as a positive control, while PBS as negative control. All sample were prepared in triplicate. After 4 h incubation at 37 °C, the non-lysed RBCs were removed from the samples through centrifugation (700g for 10 min). 100 μL of the supernatant were collected in a 96 MW and analyzed spectrophotometrically at 540 nm to quantify the hemolysis. The percentage hemolysis was determined by the following equation,

Hemolysis % = (Abs – Ass0) *100 (Abs max-Abs0)

where Abs, Ab0 and Abmax are the absorbance of: test samples, negative control (PBS) and positive control (Triton-X 100), respectively.

The level and activation state of cells involved in inflammatory response: granulocytes and monocytes/macrophages were assed in the whole blood samples challenged by increasing concentrations (0.1-100 μg/mL) of tested compounds by flow cytometry.

Leukocyte levels. 100 μL of whole blood was added to flow cytometry tube and challenged with increasing concentrations of tested compounds (0.1-100 μg/mL) or PBS as negative control. After 4 hours of incubation at 37 °C, samples were labelled anti-human CD45, anti-human CD14, anti-human CD3 conjugated antibodies for 30 minutes in the dark at RT and washed twice. Red blood cells were lysed using Lysing solution. After wash cell pellets were resuspended in 500 μL of PBS, counter stained with propidium iodide and analyzed by flow cytometry.

Statistical analysis. Flow cytometric and ELISA data were statistically analyzed using GraphPad Prism 9.00. The one-way analysis of variance ANOVA (multiple comparisons) was used for statistical analysis, with the level of significance set at *P*<0.05.

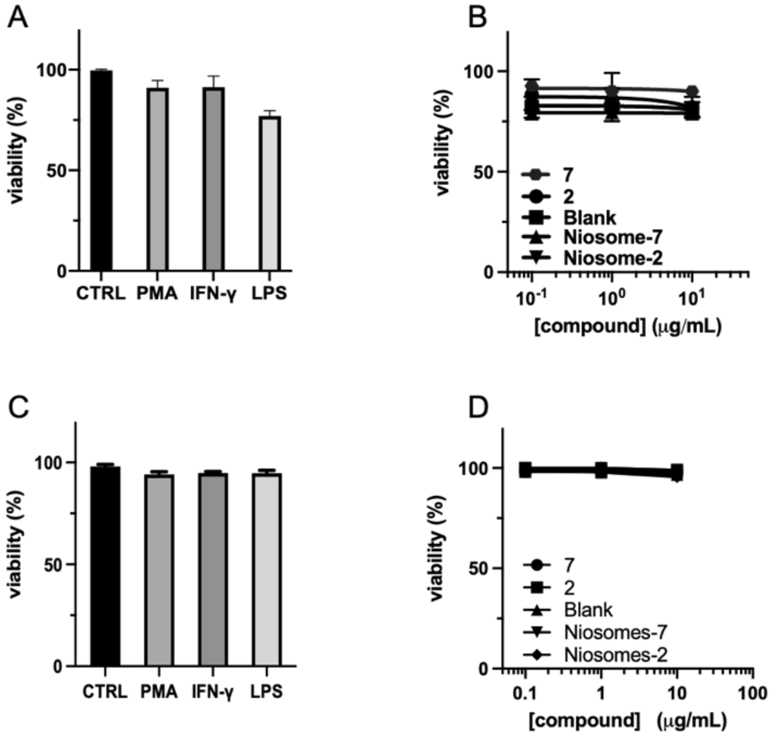

Figure S3. Effects of test compounds on THP1 and U937 cell viability. Viability of THP1(A) and U937 cells (C) treated (24 h) respectively with 150 nM or 100 nM of PMA, plus 0.5 μg/mL of LPS or 20 ng/mL of IFN-γ. Viability of THP-1 (B) and U937 (D) derived M0 treated with increasing concentrations (0.1-10 μg/mL) of glycolipids **2** or **7**, raw niosomes (blank), niosome loaded with glycolipid **7** (niosomes-**7**) and niosomes loaded with glycolipid **2** (niosomes-**2**) for 24h. Results represent mean ± SEM of at least three independent experiments.

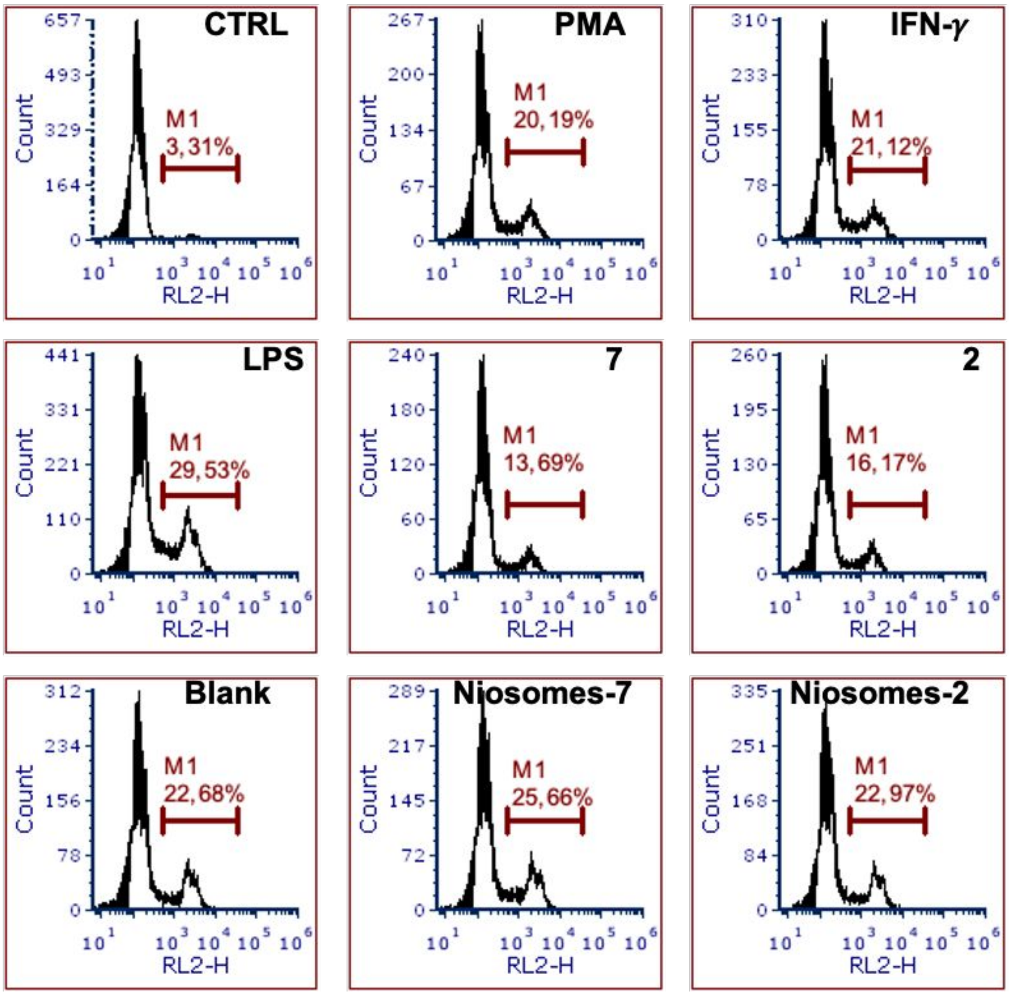

Figure S4 Representative histograms of propidium iodide fluorescence in THP1 cells treated 24h with 150 nM of PMA and of M0 treated (24h) with 0.5 mg/mL of LPS or 20 ng/mL of IFN-g or 1 mg/mL of tested compounds.(5) At the end of treatment cells were harvested labelled with propidium iodide and analyzed by FACS.

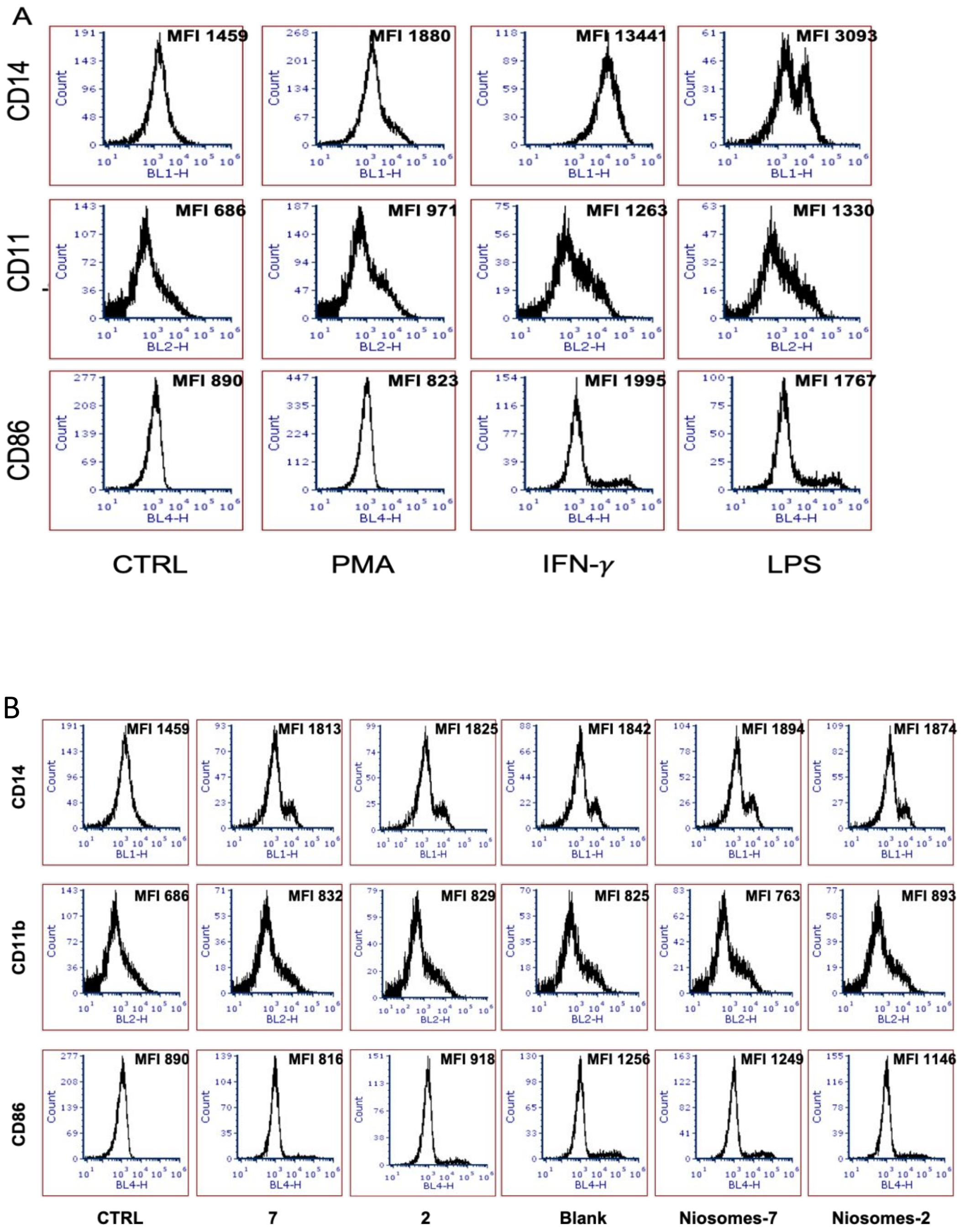

Figure S5: Surface expression markers on undifferentiated/differentiated THP1 cells. representative flow cytometry histograms showing CD14, CD11b, and CD86 expression on THP1 cells treated 24h with 150nM of PMA and of M0 treated (24h) with 0.5 mg/mL of LPS, 20 ng/mL of IFN-g (A), **7**, **2**, blank, niosomes-**7** and niosomes-**2** (B).

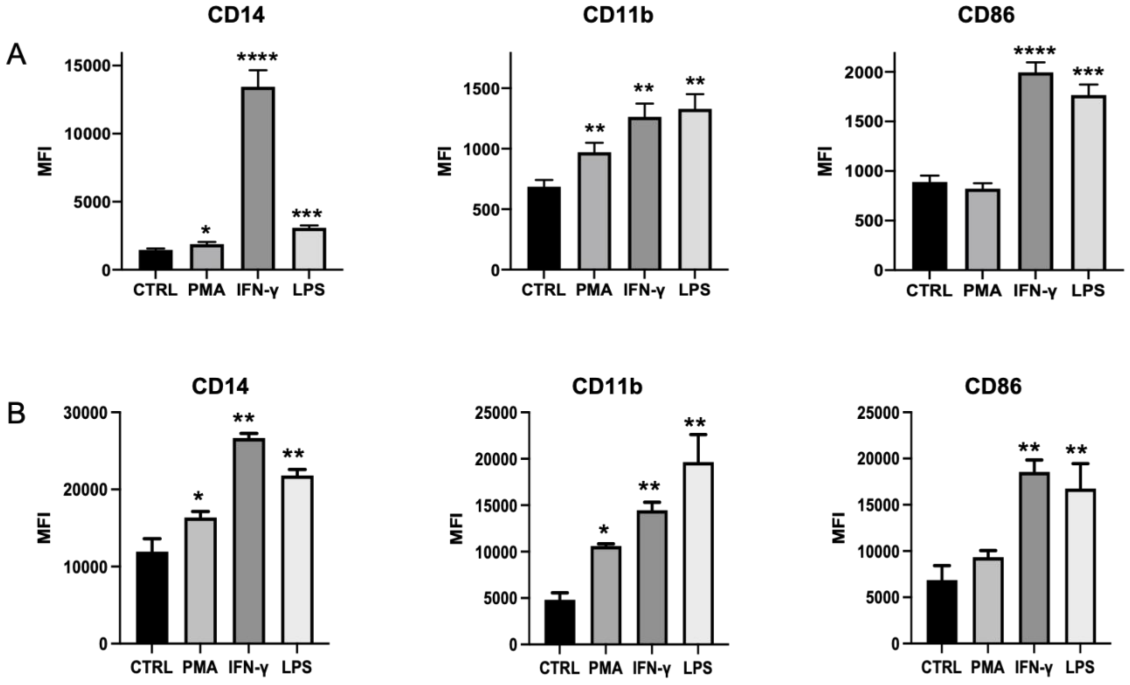

Figure S6: Surface expression markers on undifferentiated/differentiated THP1 and U937 cells. Level of expression of CD14, CD11b, and CD86 on THP1 (A) or U937 (B) cells treated 24h with 150 or 100 nM respectively, of PMA and of M0 treated (24h) with 0.5 mg/mL of LPS or 20 ng/mL of IFN-g. Results represent mean ± SEM of at least three independent experiments.

References

(1) Maestrelli, F.; Landucci, E.; De Luca, E.; Nerli, G.; Bergonzi, M. C.; Piazzini, V.; Pellegrini-Giampietro, D. E.; Gullo, F.; Becchetti, A.; Tadini-Buoninsegni, F.; et al. Niosomal Formulation of a Lipoyl-Carnosine Derivative Targeting TRPA1 Channels in Brain. *Pharmaceutics* **2019**, *11* (12), 669.

(2) Bragagni, M.; Mennini, N.; Ghelardini, C.; Mura, P. Development and Characterization of Niosomal Formulations of Doxorubicin Aimed at Brain Targeting. *J. Pharm. Pharm. Sci.* **2012**, *15* (1), 184.

(3) Ingallina, C.; Rinaldi, F.; Bogni, A.; Ponti, J.; Passeri, D.; Reggente, M.; Rossi, M.; Kinsner-Ovaskainen, A.; Mehn, D.; Rossi, F.; et al. Niosomal Approach to Brain Delivery: Development, Characterization and in Vitro Toxicological Studies. *Int. J. Pharm.* **2016**, *511* (2), 969–982.

(4) Zaccai, G. How Soft Is a Protein? A Protein Dynamics Force Constant Measured by Neutron Scattering. *Science (80-.).* **2000**, *288* (5471), 1604–1607.

(5) Bertani, F. et al Scientific report, **2017**, *7,* 8965.
